# Natural course of health and well-being in non-hospitalised children and young people after testing for SARS-CoV-2: a prospective follow-up study over 12 months

**DOI:** 10.1016/j.lanepe.2022.100554

**Published:** 2022-12-05

**Authors:** Snehal M. Pinto Pereira, Roz Shafran, Manjula D. Nugawela, Laura Panagi, Dougal Hargreaves, Shamez N. Ladhani, Sophie D. Bennett, Trudie Chalder, Emma Dalrymple, Tamsin Ford, Isobel Heyman, Kelsey McOwat, Natalia K. Rojas, Kishan Sharma, Ruth Simmons, Simon R. White, Terence Stephenson

**Affiliations:** a Division of Surgery & Interventional Science, Faculty of Medical Sciences, University College London, WC1E 6BT, UK; b UCL Great Ormond Street Institute of Child Health, 30 Guilford Street, London, WC1N 1EH, UK; c Department of Psychiatry, University of Cambridge, Hershel Smith Building Cambridge Biomedical Campus, CB2 0SZ, UK; d Mohn Centre for Children's Health & Wellbeing, School of Public Health, Imperial College London, UK; e Immunisation Department, Public Health England, 61 Colindale Avenue, London, NW9 5EQ, UK; f Paediatric Infectious Diseases Research Group, St. George's University of London, Cranmer Terrace, London, SW17 0RE, UK; g Department of Psychological Medicine, Institute of Psychiatry, Psychology and Neuroscience, King's College London, De’Crespigny Park, London, SE5 8AF, UK; h Medical Research Council Biostatistics Unit, University of Cambridge, East Forvie Building, Cambridge Biomedical Campus, CB2 0SR, UK; i Division of Neuroscience & Experimental Psychology, University of Manchester, UK

**Keywords:** Long COVID, Symptoms, Well-being, Children and young people, Longitudinal, CYP, Children and young people, UKHSA, United Kingdom Health Security Agency, IQR, Interquartile range

## Abstract

**Background:**

Despite high numbers of children and young people (CYP) having acute COVID, there has been no prospective follow-up of CYP to establish the pattern of health and well-being over a year following infection.

**Methods:**

A non-hospitalised, national sample of 5086 (2909 SARS-COV-2 Positive; 2177 SARS-COV-2 Negative at baseline) CYP aged 11–17 completed questionnaires 6- and 12-months after PCR-tests between October 2020 and March 2021 confirming SARS-CoV-2 infection (excluding CYP with subsequent (re)infections). SARS-COV-2 Positive CYP was compared to age, sex and geographically-matched test-negative CYP.

**Findings:**

Ten of 21 symptoms had a prevalence less than 10% at baseline, 6- and 12-months post-test in both test-positives and test-negatives. Of the other 11 symptoms, in test-positives who had these at baseline, the prevalence of all symptoms declined greatly by 12-months. For CYP first describing one of these at 6-months, there was a decline in prevalence by 12-months. The overall prevalence of 9 of 11 symptoms declined by 12-months. As many CYP first described shortness of breath and tiredness at either 6- or 12-months, the overall prevalence of these two symptoms in test-positives appeared to increase by 6-months and increase further by 12-months. However, within-individual examination demonstrated that the prevalence of shortness of breath and tiredness actually declined in those first describing these two symptoms at either baseline or 6-months. This pattern was also evident for these two symptoms in test-negatives. Similar patterns were observed for validated measures of poor quality of life, emotional and behavioural difficulties, poor well-being and fatigue. Moreover, broadly similar patterns and results were noted for the sub-sample (N = 1808) that had data at baseline, 3-, 6- and 12-months post-test.

**Interpretation:**

In CYP, the prevalence of adverse symptoms reported at the time of a positive PCR-test declined over 12-months. Some test-positives and test-negatives reported adverse symptoms for the first time at six- and 12-months post-test, particularly tiredness, shortness of breath, poor quality of life, poor well-being and fatigue suggesting they are likely to be caused by multiple factors.

**Funding:**

10.13039/100012411NIHR/10.13039/100014013UKRI (ref: COVLT0022).

**Research in context**

**Evidence before this study**

We previously published health and well-being profiles of children and young people (CYP) three months after a positive or negative PCR test for SARS-COV-2. There are now a number of cross-sectional surveys from several countries but we are unaware of any published studies (including from those identified in our systematic review) on individual-level prospective follow-ups of CYP with confirmed SARS-CoV-2 infection and matched SARS-CoV-2 test-negatives to assess the natural course of post-COVID-19 health and well-being in individuals. Here, we describe the self-reported health and well-being profiles on a matched cohort of individuals at both six and twelve months after a positive or negative SARS-CoV-2 PCR test.

**Added value of this study**

This is a unique population-based cohort study of CYP with PCR-confirmed SARS-CoV-2 infection status where health and well-being are reported by CYP themselves. Importantly, there is a matched test-negative group of CYP who have lived through the ‘long pandemic’ and who have never tested positive for SARS-CoV-2 (determined by PCR test and self-report). Participants were recruited nationally. We evaluated the prevalence of health and well-being in both test negative and positive groups. We tracked the adverse symptoms in this cohort longitudinally over a 12-month period and show that the prevalence of adverse symptoms reported at the time of a positive PCR-test declined over 12-months. However, new adverse symptoms were reported six- and 12-months post-test by both test-positives and test-negatives, particularly tiredness, shortness of breath, poor quality of life, poor well-being and fatigue. Our study demonstrates the added value of longitudinal, individual-level follow-up studies.

**Implications of all the available evidence**

This unique study finds that in most CYP, specific adverse symptoms reported at testing and 6-months later had resolved by 12-months, although in a minority they were persistent, and that new-onset had emerged. If we had simply looked at cross-sectional prevalence of adverse symptoms at testing, 6-months and 12-months, as is commonly done in other studies, it would have appeared as if the prevalence of specific common post-COVID-symptoms stayed largely stable, or increased, over time. However, we show that this is not the case. The new-onset adverse symptoms arising 6- or 12-months after initial viral infection should not exclusively be viewed as new long COVID symptoms as a consequence of the initial SARS-COV-2 infection. Rather, these adverse symptoms should be seen in the wider context of health and well-being in the general adolescent population. Recent reviews of Long COVID in CYP indicate that higher quality studies are needed and that a consistent definition of Long COVID is required; our research goes one step further and indicates that studies with repeat measurement on the same CYP are needed to track individual trajectories and not simply report repeat cross-sectional prevalence's of symptoms over time.

## Introduction

For most children and young people (CYP), SARS-CoV-2 infection has been asymptomatic or a mild illness^1^ compared to adults.^2^ However, as the cumulative incidence of infection in CYP increases, the incidence of post-COVID sequelae has become a growing concern. Long COVID (post-COVID-19 condition), has a debilitating impact on some CYP but little is known about the frequency, distribution or duration of poor health and well-being after SARS-CoV-2 infection in CYP.^3^

In our systematic review^4^ of 22 studies, the most common symptoms in CYP at 3 months were fatigue, insomnia, loss of smell, and headaches; additional reported symptoms included anxiety, low mood and ‘brain fog’. Only five studies identified in the review had a negative test control group to disentangle the effects of infection from living through a pandemic. There is considerable variation in the published literature on the natural history of long-term poor health and well-being associated with SARS-CoV-2 infection and even less data on the associated burden beyond 3 months in CYP.^1^^,^^5^, ^6^, ^7^, ^8^, ^9^, ^10^

The CLoCk study is the largest national, matched longitudinal cohort study of CYP in England,^11^ whereby non-hospitalised teenagers self-report on post-COVID-19 health and well-being after PCR-confirmed SARS-CoV-2 infection compared to SARS-CoV-2 PCR-negative CYP.^11^^,^^12^ At 3-months post-test, among a subsample of 6084 participants,^12^ 66.5% of test-positives and 53.3% of test-negatives had any symptoms. In contrast, at testing, 35.4% test-positives and 8.3% test-negatives reported any symptoms. This paradoxical increase in symptoms from time of testing to 3 months post-test, potentially due to self-selection, made it essential to follow the cohort longitudinally for 12 months after PCR-testing to understand the within-individual trajectory of health and well-being over time. We therefore collected longitudinal information on a larger group of CYP at 6- and 12-months post-test and here we describe the within-individual variation in health and well-being 6- and 12-months after testing.

## Methods

The CLoCk study, described in detail elsewhere,^11^ is a cohort study of SARS-CoV-2 PCR-positive CYP aged 11–17 years, matched by month of test, age, sex, and geographical area to SARS-CoV-2 test-negative CYP using the national SARS-CoV-2 testing dataset held by United Kingdom Health Security Agency (UKHSA).

**Fig. 1:**
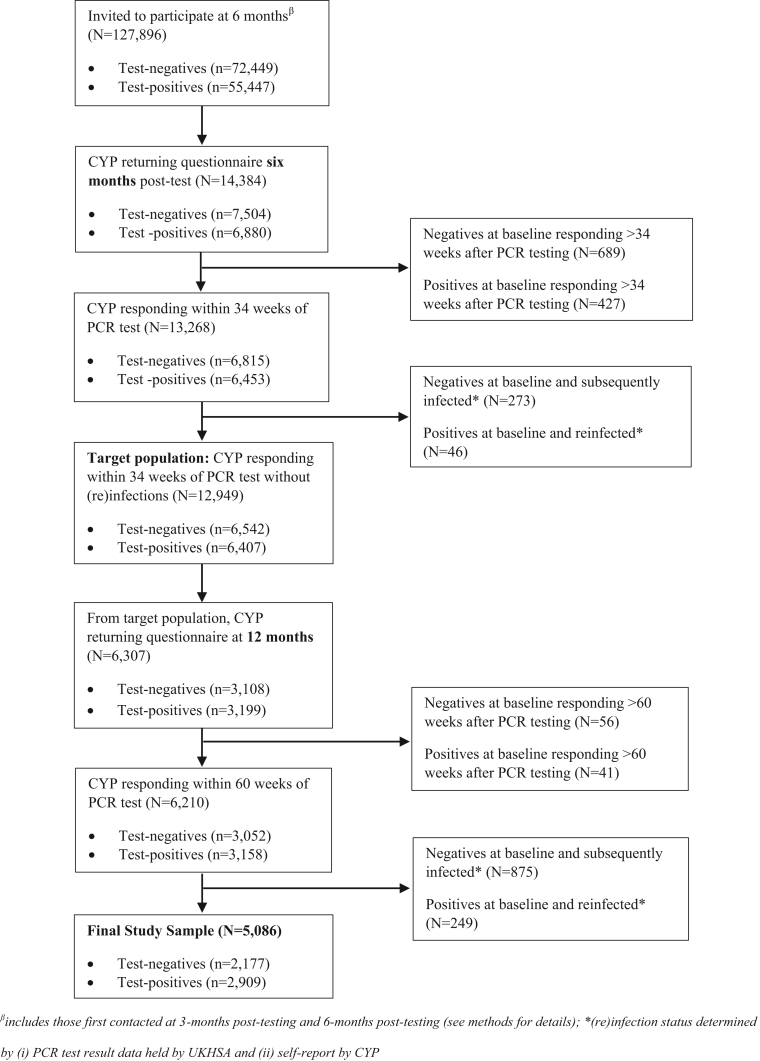
Participant flow diagram.

The study has recruited >30,000 CYP in total with a goal of collecting data for 24-months after a SARS-CoV-2 PCR test taken between September 2020 and March 2021. Depending on the month of test, for some participants we collect data at 3-, 6-, 12- and 24-months post-test; for others 6-, 12- and 24-months post-test; and for some 12- and 24-months post-test.^11^ Here we report on data acquired on the *same* CYP at 6-months and 12-months after PCR-testing (we also do a sensitivity analysis on the sub-sample of CYP with data at 3-, 6- and 12-months after PCR-testing, see below). Following informed consent, at first contact included CYP completed an online questionnaire about their health at the time of their PCR test (i.e. baseline at 0-months) and at the time of completing the questionnaire (approximately 3- or 6-months after their PCR test). CYP completed subsequent questionnaires at 6-months (for the sub-sample first contacted at 3-months) and 12-months, that asked about their health and well-being at the time of the questionnaire. Questionnaires were filled in by the CYP themselves, however, a carer could assist younger CYP and those with special educational needs or disability. After excluding test-positives who were reinfected and test-negatives who were infected after baseline testing (determined by PCR test results held by UKHSA and self-report of whether (or not) the CYP ever had a positive COVID-19 test, including Lateral Flow Tests), 12,949 participants who responded at 6 months post-test were included (Fig. 1). This group was approached again at 12 months post-test, and after additional exclusions, the final analytical sample comprised 5086 CYP (2909 test-positives, 2177 test-negatives, see Fig. 1).

In this analytical sample, 1934 of 2909 (66.5%) test-positive and 1445 of 2177 (66.4%) test-negative CYP had received a COVID-19 vaccine between 6- and 12-months follow-up. Sixty-two of 2909 (2.1%) SARS-CoV-2 PCR-positive CYP attended hospital during the 12-month follow-up period, including 16 who were hospitalised overnight.

### Measures

The measures included demographics, elements of the International Severe Acute Respiratory and emerging Infection Consortium (ISARIC) Paediatric COVID-19 questionnaire,^13^ and the recent Mental Health of Children and Young people in England surveys.^14^ Based on the ISARIC Paediatric Working Group, we included 21 symptoms^12^ and validated instruments for loneliness (the adapted 3-item UCLA Loneliness Scale),^15^^,^^16^ mental health and wellbeing (Strengths and Difficulties Questionnaire,^17^ Short Warwick Edinburgh Mental Wellbeing Scale^18^^,^^19^), the Chalder Fatigue Scale^20^ and the EQ-5D-Y^21^ as a measure of quality of life and functioning (see details in Supplementary Table S1). The questionnaires were largely unchanged between the 6- and 12-month follow-up (see Supplementary text A for details).

We operationalised the established Delphi research definition of long COVID^22^ as having at least one of the 21 reported symptoms and experiencing more than minimal problems on any one of the five EQ-5D-Y questions (see Supplementary Table S1). The Delphi research definition requires laboratory confirmation of SARS-COV-2 infection but of course that was not required when assessing how many test-negatives would also have met this definition.

### Statistical methods

We first assessed the representativeness of our analytic sample by comparing their demographic characteristics (sex, age at testing, region of residence, and Index of Multiple Deprivation) to the target population invited 6-months post-test. Second, we described the prevalence of each of the health and well-being measures in two ways: (a) we tabulated the prevalence in CYP who had an adverse symptom never, once, twice or thrice and assessed whether the prevalence differed by SARS-CoV-2 PCR status; (b) taking into consideration the temporal nature of the data and the repeated measures on the same CYP over time, we generate stacked bar charts that show the distribution of health and well-being across the three time-points and indicate when the adverse symptom was first reported. Both analyses were stratified by SARS-CoV-2 status.

### Sensitivity and exploratory analyses

We did one sensitivity and one exploratory analysis.

Sensitivity analysis: as indicated above, information was collected on a sub-sample 3-months post-test; the above-described analysis was therefore repeated on the smaller sample with data at 0-, 3-, 6-, and 12-months post-test.

Exploratory analysis: although not designed to answer questions regarding school attendance after COVID-19 infection, this information is needed to guide education support strategies. Thus, we explored self-reported school absence data in CLoCk participants 6-months after initial PCR-testing.

### Role of funding source

The 10.13039/501100000276Department of Health and Social Care, as the National Institute for Health Research (NIHR), and 10.13039/100014013UK Research & Innovation (UKRI) awarded grant COVLT0022 but were not involved in study design, data collection, analysis, interpretation or writing.

## Results

The 6- and 12-month follow-up questionnaires were returned at a median of 27.7 [IQR: 26.1, 29.6] and 52.1 [IQR: 50.7, 54.1] weeks after testing, respectively. In total, 2909 of 6407 (45.4%) SARS-COV-2 positive and 2177 of 6542 (33.3%) SARS-COV-2 negative CYP who responded at 6-months also responded at 12-months. Both test-positives and test-negatives in the analytical sample were broadly similar to the target population responding at 6 months, albeit test-negatives were slightly older than test-positives (Table 1).

**Table 1:** Comparison of target population to analytic sample; and characteristics of children and young people (CYP) in analytic sample by baseline PCR-test result: N (%).

Characteristic	Target population of CYP who responded at 6 months post-test	CYP in analytic sample (responding at 6- and 12-months post-test)	SARS-CoV-2 Negative	SARS-CoV-2 Positive
N	12,949	5086	2177	2909
SARS-CoV-2				
Negative	6542 (50.5)	2177 (42.8)	2177 (100.0)	0 (0.0)
Positive	6407 (49.5)	2909 (57.2)	0 (0.0)	2909 (100.0)
Age at testing (years)				
11–14	5573 (43.0)	2047 (40.2)	806 (37.0)	1241 (42.7)
15–17	7376 (56.9)	3039 (59.8)	1371 (63.0)	1668 (57.3)
Sex				
Male	4845 (37.4)	1785 (35.1)	765 (35.1)	1020 (35.1)
Female	8104 (62.6)	3301 (64.9)	1412 (64.9)	1889 (64.9)
Ethnicity				
White	10,004 (77.3)	3958 (77.8)	1680 (77.2)	2278 (78.3)
Asian or Asian British	1774 (13.7)	694 (13.7)	304 (14.0)	390 (13.4)
Mixed	570 (4.4)	228 (4.5)	109 (5.0)	119 (4.1)
Black, African, or Caribbean	325 (2.5)	126 (2.5)	56 (2.6)	70 (2.4)
Other	205 (1.6)	57 (1.1)	20 (0.9)	37 (1.3)
Prefer not to say	71 (0.5)	23 (0.4)	8 (0.4)	15 (0.5)
IMD^a^				
1 (most deprived)	2554 (19.7)	894 (17.6)	390 (17.9)	504 (17.3)
2	2344 (18.1)	903 (17.7)	384 (17.6)	519 (17.8)
3	2340 (18.1)	953 (18.7)	425 (19.5)	528 (18.2)
4	2710 (20.9)	1104 (21.7)	474 (21.8)	630 (21.7)
5 (least deprived)	3001 (23.2)	1232 (24.2)	504 (23.2)	728 (25.0)
Region				
East Midlands	1353 (10.4)	531 (10.4)	239 (11.0)	292 (10.1)
East of England	1391 (10.7)	599 (11.8)	269 (12.4)	330 (11.3)
London	1549 (12.0)	613 (12.1)	295 (13.6)	318 (10.9)
North East	786 (6.1)	290 (5.7)	112 (5.1)	178 (6.1)
North West	1901 (14.7)	713 (14.0)	282 (13.0)	431 (14.8)
South East	1775 (13.7)	751 (14.8)	321 (14.7)	430 (14.8)
South West	987 (7.6)	402 (7.9)	168 (7.7)	234 (8.1)
West Midlands	1724 (13.3)	672 (13.2)	294 (13.5)	378 (13.0)
Yorkshire and The Humber	1483 (11.5)	515 (10.1)	197 (9.0)	318 (10.9)

a Index of Multiple Deprivation (IMD), derived from the CYP's lower super output area (a small local area level based geographic hierarchy), was used as a proxy for socio-economic status. We used IMD quintiles from most (quintile 1) to least (quintile 5) deprived.

### Symptom profiles at baseline, 6- and 12-months post-test

The prevalence of CYP reporting the same symptom never, once, twice or at all three time points is shown in Supplementary Table S2. Among the test-positives, 10.9% reported fatigue, 4.4% reported shortness of breath, 3.3% loss of smell or taste, 1.7% dizziness or light-headedness, and 1.1% described skipping meals at all three time points. The other 16 symptoms affected less than 1% of test-positives at all three time points. Among test-negatives, 1.2% reported fatigue at all three time points. The other 20 symptoms were reported by less than 1% of test-negatives at all three time points. Thus, the distribution of symptom prevalence differed by SARS-CoV-2 PCR status (p ≤ 0.004) except for experiencing sores or blisters on feet (p = 0.064).

**Fig. 2:**
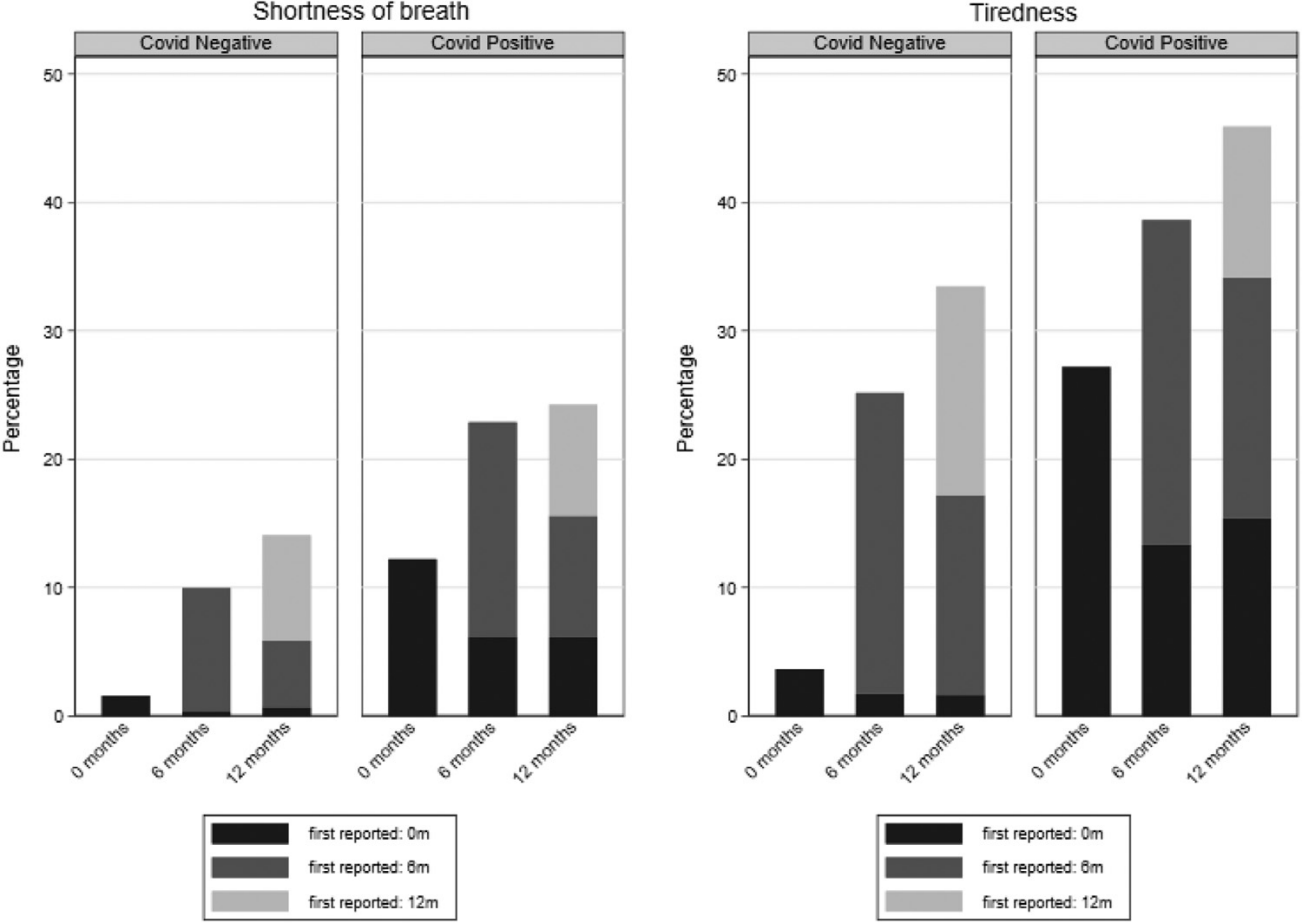
Symptoms with overall prevalence increasing from baseline to 12 months and remaining high.

When assessing overall prevalence at the three time points in more detail, we categorised symptom patterns into three broad groups: (i) Ten symptoms with low overall prevalence (less than 10%) at all three time points in both test-negatives and test-positives (Supplementary Fig. S1), (ii) Nine symptoms where the overall prevalence declined from baseline to 12 months post-test in test-positives (Supplementary Fig. S2) and fluctuated variably but at low prevalence in test-negatives; and (iii) Two symptoms with overall prevalence increasing from baseline to 12-months and remaining high in both test-negatives and test-positives (Fig. 2).

When examining within-individual change in symptom profiles, the prevalence of the 11 more common symptoms at baseline (i.e., baseline prevalence >10%) declined greatly by 12-months, in the test-positives (Supplementary Fig. S2 and Fig. 2). For CYP who first describe one of these symptoms at 6-months, again there is a decline in prevalence by 12 months (Supplementary Fig. S2 and Fig. 2). In keeping with this, the overall prevalence (i.e., total height of bar charts) for 9 out of 11 symptoms declined by 12-months (p ≤ 0.2 for difference between proportion of CYP with symptoms at baseline and 12-months post-test in test-positives; Supplementary Fig. S2). However, for two symptoms, shortness of breath and tiredness, the overall prevalence in test-positives increased by 6-months and increased further by 12-months, because large numbers of CYP first describe these symptoms at either 6-months or 12-months; this pattern was also observed for these two symptoms among test-negatives (Fig. 2). At 12-months, the difference in prevalence between test-positives and test-negatives for these two symptoms, varied by when the symptom was first reported. For example, for test-positives and test-negatives who reported shortness of breath for the first time at baseline (time of PCR test), the difference in prevalence of shortness of breath at 12 months between the test-positives and test-negatives was 5.43% (95% CI:4.49%, 6.36%); the difference in prevalence among those reporting shortness of breath for the first time at 12 months was 0.44% (95% CI:-1.10%,1.98%), Table 2.

**Table 2:** Difference in prevalence of selected^a^ health and well-being measures between test-positives and test-negatives at 12 months, by time symptom first reported.

Prevalence difference at 12 months in test-positives and test-negatives (95% CI)	
Shortness of breath	
First reported at:	
0 months	5.43 (4.49, 6.36)
6 months	4.30 (2.89, 5.71)
12 months	0.44 (−1.10, 1.98)
Tiredness	
First reported at:	
0 months	13.75 (12.33, 15.16)
6 months	3.21 (1.12, 5.28)
12 months	−4.50 (−6.44, −2.56)
Having pain or discomfort^b^	
First reported at:	
0 months	−1.79 (−3.19, −0.38)
6 months	1.71 (0.67, 2.75)
12 months	0.21 (−1.37, 1.80)
Difficulty doing usual activities^b^	
First reported at:	
0 months	−1.52 (−2.64, −0.39)
6 months	2.23 (1.26, 3.20)
12 months	1.08 (- 0.28, 2.44)
Mental health^c^	
High/very high total difficulties	
First reported at:	
6 months	−1.98 (−3.79, −0.16)
12 months	0.17 (−1.31, 1.64)
High/very high emotional difficulties	
First reported at:	
6 months	−1.61 (−3.77, 0.55)
12 months	−0.39 (−2.13, 1.34)
High/very high hyperactivity	
First reported at:	
6 months	−0.40 (−2.19, 1.38)
12 months	−0.07 (−1.52, 1.38)
High/very high peer difficulties	
First reported at:	
6 months	−3.02 (−4.95, −1.10)
12 months	−1.44 (−3.09, 0.20)
High/very high impact	
First reported at:	
6 months	−2.51 (−4.41, −0.60)
12 months	−0.18 (−1.84, 1.48)
Poor well-being^c^	
First reported at:	
6 months	−3.30 (−5.68, −0.92)
12 months	1.61 (−0.39, 3.60)
Severe fatigue^c^	
First reported at:	
6 months	4.30 (1.94, 6.65)
12 months	−1.49 (−3.43, 0.46)
Long COVID^c^	
First reported at:	
6 months	6.25 (4.42, 8.07)
12 months	−0.30 (−2.05, 1.45)

Calculated as: % with symptom at 12 months in test-positives - % with symptom at 12 months in test-negatives.

a Selected based on (i) overall prevalence increasing from baseline to 12 months and (ii) prevalence in test-positives >10% at least twice.

b From EQ-5D-Y.

c Using the Strengths and Difficulties Questionnaire, Short Warwick Edinburgh Mental Wellbeing Scale, Chalder Fatigue Scale and operationalisation of the Delphi definition of Long COVID respectively (see Supplementary Table S1 for details).

### Quality of life/functioning and Loneliness profiles at baseline, 6- and 12-months post-test

**Fig. 3:**
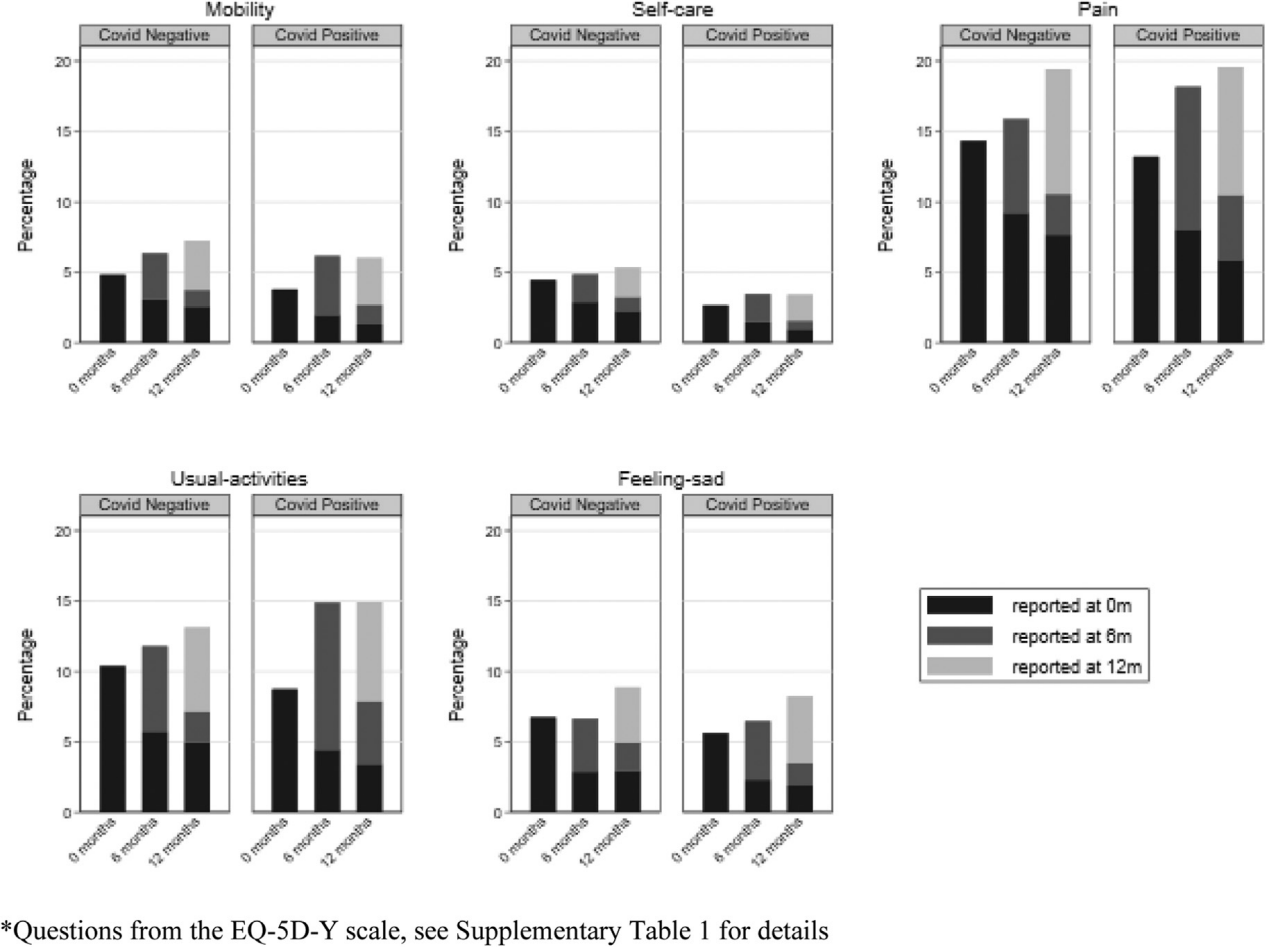
Prevalence of poor quality of life and functioning∗ over a 12-month period in test-positives and test-negatives.

**Fig. 4:**
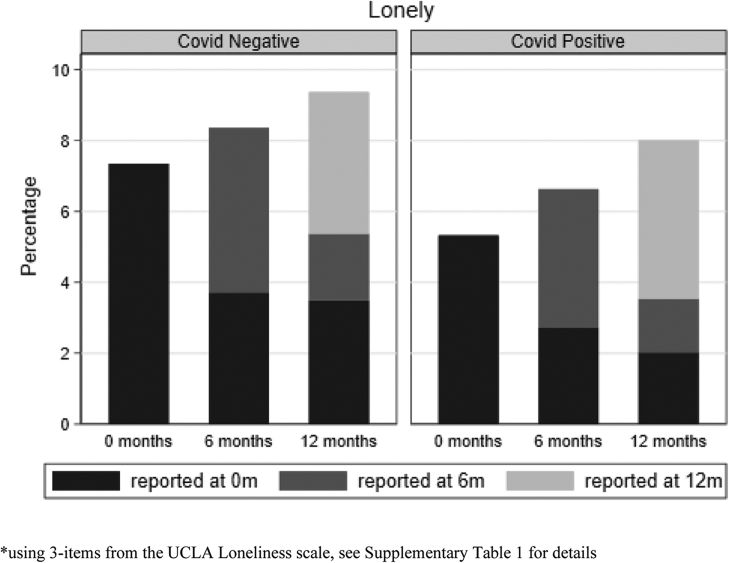
Prevalence of loneliness∗ over a 12-month period in test-positives and test-negatives.

The overall prevalence of problems with mobility, self-care, feeling sad (EQ-5D-Y) or lonely (adapted 3-item UCLA Loneliness scale) was low (less than 10%) at all three time points in both test-negatives and test-positives (Fig. 3, Fig. 4). Problems with doing usual activities and having pain followed similar patterns to those observed for shortness of breath and tiredness (i.e., overall prevalence in test-positives increased by 6-months and generally increased further by 12-months, because large numbers of CYP first report these conditions at either 6-months or 12-months; Fig. 3). However, there was little difference in the prevalence of having pain or difficulty doing usual activities between test-positives and test-negatives reporting these for the first time at 12 months (Table 2).

### Mental health, well-being, fatigue and long COVID at 6- and 12-months post-test

**Fig. 5:**
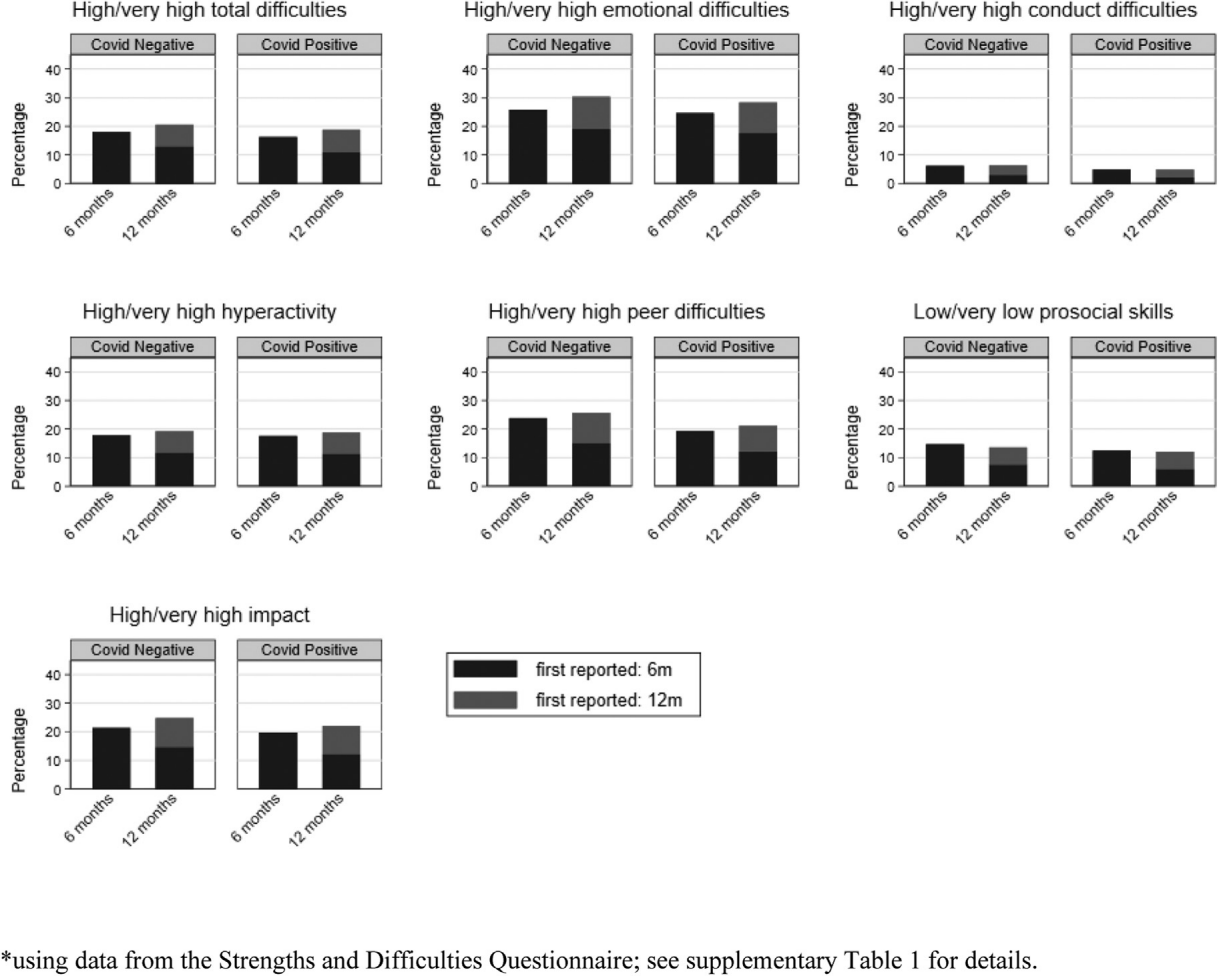
Prevalence of emotional and behavioural difficulties∗ over a 12-month period in test-positives and test-negatives.

**Fig. 6:**
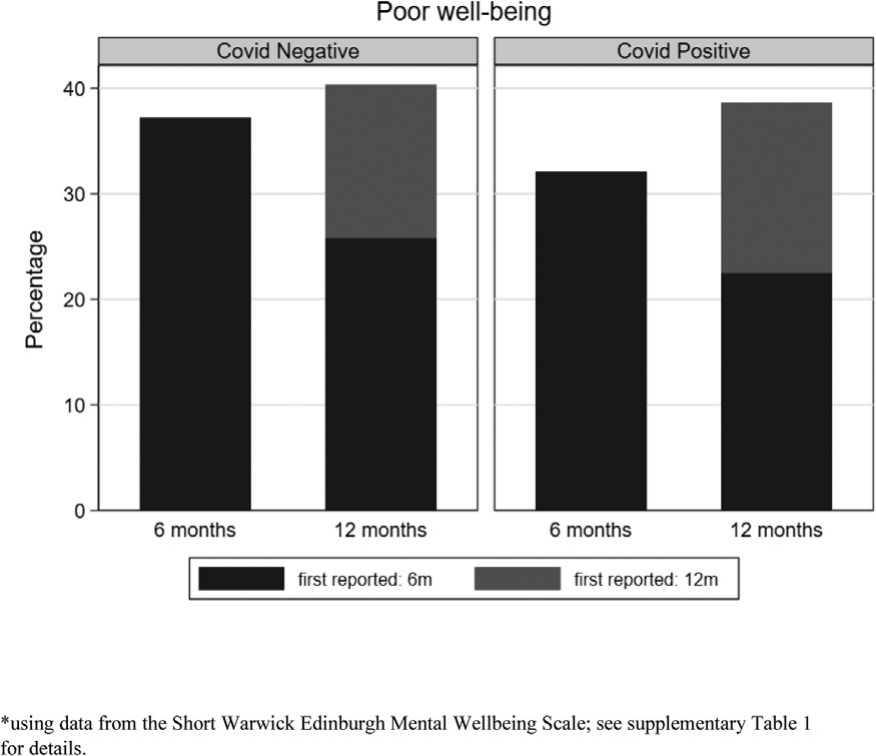
Prevalence of poor well-being∗ over a 12-month period in test-positives and test-negatives.

**Fig. 7:**
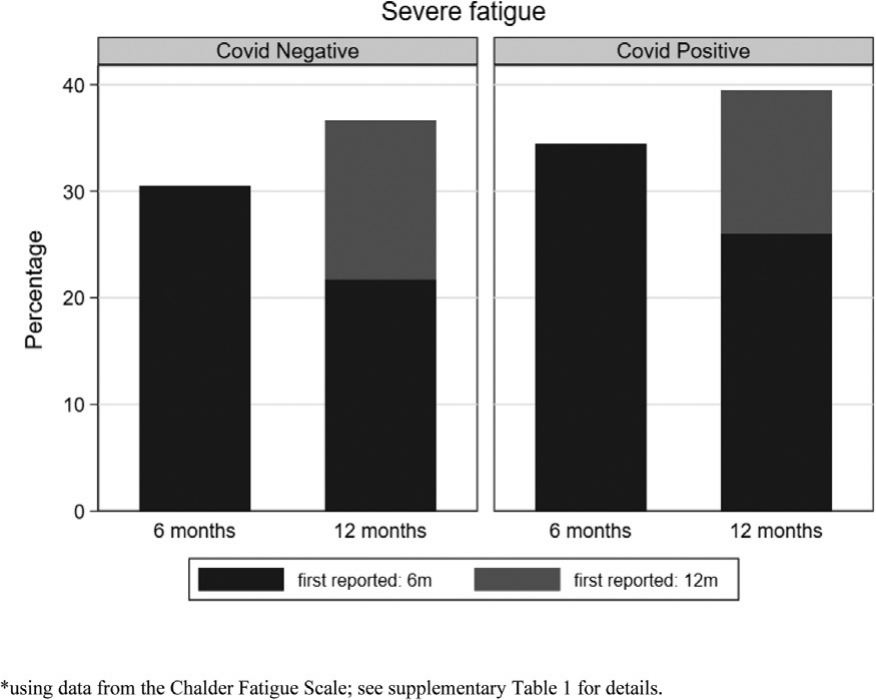
Prevalence of severe fatigue∗ over a 12-month period in test-positives and test-negatives.

**Fig. 8:**
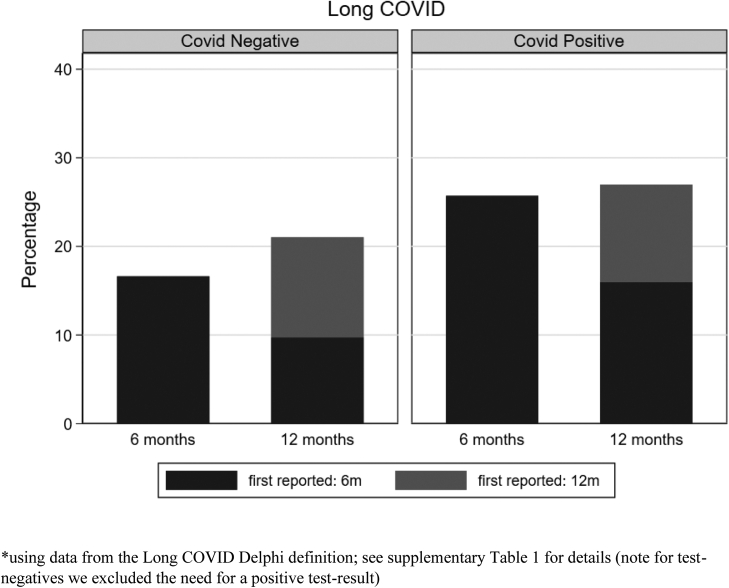
Prevalence of long COVID∗ over a 12-month period in test-positives and test-negatives.

The overall prevalence of conduct difficulties was low at 6- and 12-months post-test and for low prosocial skills, decreased slightly (Fig. 5). For the other five adverse outcomes from the Strengths and Difficulties Questionnaire, between 6- and 12-months the overall prevalence increased slightly (Fig. 5) and there was little difference in the prevalence of these measures between test-positives and test-negatives reporting them for the first time at 12-months (Table 2). The overall and within-individual prevalence patterns of poor well-being (Fig. 6), fatigue (Fig. 7) and Long COVID (Fig. 8) were broadly similar, and again there was little difference in the prevalence of these measures between test-positives and test-negatives reporting them for the first time at 12-months (Table 2).

### Sensitivity and exploratory analysis

In the sub-sample with data collected at 3-months post-test (N = 1808, Supplementary Fig. S3), broadly similar patterns and results were observed to those reported above (Supplementary Figs. S4–S12; Supplementary Tables S3 and S4).

In exploratory analysis, we found that among symptomatic CPY, school absence (≥1 day) 6-months post-test was less common in SARS-CoV-2 PCR-positive participants than PCR-negative participants, but a higher proportion reported *extended* school absence of >10 days (p < 0.001). In contrast, CYP who were asymptomatic reported lower absence rates 6-months post-test (Supplementary text B; Supplementary Fig. S13; Supplementary Table S5).

## Discussion

We report here the prevalence of health and well-being at 6- and 12-months after laboratory-confirmed SARS-COV-2 infection, which we believe to be the only longitudinal follow-up in CYP in a matched cohort. The results show that aggregating across all three time points, adverse symptoms were generally more common in test-positive compared to test-negative CYP (the ‘Never had adverse symptoms’ columns in Supplementary Table S2). The majority of test-positive CYP who had a particular adverse symptom at testing were free from that symptom at both 6- and 12-months post-test, demonstrating that these symptoms generally improved over time. Additionally, most CYP who first developed a particular symptom 6-months after their positive (or negative) PCR-test did not report that symptom at 12-months. We also found in the sub-sample with data collected at 3-, 6- and 12-months post-test, broadly similar patterns and results.

The symptom prevalence during acute SARS-CoV-2 infection among test-positive CYP was similar in our cohort when compared to those reported in other adolescent cohorts,^23^^,^^24^ indicating that our analytical cohort is representative of CYP in general. The very low prevalence of loss of smell/taste among test-negatives – both at testing and over the 12-month follow-up period, also provides some reassurance of a low rate of unconfirmed SARS-CoV-2 infections in the test-negative group, although we acknowledge (re)infections may have gone undetected.

For two symptoms, (shortness of breath and tiredness) as well as measures of poor quality of life (in particular having pain and problems doing usual activities), poor well-being and fatigue, the overall prevalence in test-positives increased over time. Importantly, our within-individual exploration demonstrates that the prevalence actually declined in those who first described these adverse symptoms at either baseline or 6-months. Differences in the prevalence of these adverse symptoms between test-positives and test-negatives remained at 12-months but varied depending on when the symptom was first reported. For example, there was no difference in the prevalence of shortness of breath between test-positives and test-negatives if it was first reported at 12-months post-test. The prevalence of tiredness was (surprisingly) less common in the test-positives, if first reported at 12-months post-test. However, if either symptom was first reported at time of testing, the prevalence at 12-months was higher among test-positives by 5.4% (shortness of breath) and 13.8% (tiredness) compared to test-negatives. The broadly similar pattern of adverse health and well-being reported as new-onset at 6- and 12-months among test-positives and test-negatives highlights the non-specific nature of these symptoms and suggests that multiple aetiologies may be responsible for CYP experiencing these symptoms over time. Further studies are therefore needed to understand the cause of persistent adverse health and well-being in test-positive CYP and how they differ from test-negatives reporting the same adverse symptoms.

Our consistent and robust findings across a diverse range of health and well-being measures emphasises (i) the close relationship between physical and mental health and (ii) the value of repeated measures over time in the same individuals. Taking all the data in consideration, we found that if we had simply looked at cross-sectional prevalence's at baseline, 3- (in the sub-sample), 6- and 12-months, it would have appeared as if the prevalence of several adverse post-COVID-symptoms remained largely stable, or even increased, over time. In fact, most (but not all) CYP recovered from the adverse symptoms which they experienced at baseline and 6-months post-infection. However, the reporting of new onset of these same symptoms at 6- and 12-months follow-up by both test-positive and test-negative CYP suggests that these symptoms may be causally related to multiple factors and not just the original SARS-COV-2 infection. For example, the development of new symptoms 6- or 12-months after their SARS-COV-2 PCR-test in both test-positives and test-negatives could represent background levels of symptomatology in CYP in England. This highlights the need for appropriate control groups in long COVID studies and normative population studies of common symptoms among CYP outside of the context of a pandemic.

Similar to our within-individual findings, in adults persisting post-COVID-19 symptoms have also been shown to decline with time.^5^ Pooled prevalence data from 27 eligible research publications in adults showed the 5 most prevalent reported symptoms were fatigue, shortness of breath, muscle pain, cough and headache, overlapping with the commonest symptoms we describe in CYP in our cohort.^25^ Furthermore, in a recent review of nine UK longitudinal studies in adults, totalling over 42,000 participants, the symptoms characteristic of long COVID were similar to the commonest symptoms we describe in CYP, including fatigue, shortness of breath and muscle pain or aches, but also difficulty concentrating and chest tightness.^26^

Long-term follow-up data in CYP is sparse. A single-centre, hospital-based Australian study followed 171 CYP for 1-1.5 years after SARS-COV-2 infection and showed resolution of all symptoms.^6^ A national cohort study of 37,522 CYP with laboratory-confirmed SARS-CoV-2 infection in Denmark and a control group of 78,037 randomly selected uninfected children^9^ also reported that in most children, ‘long COVID’ symptoms resolved by 5 months. However, a large population study using nationwide registry data from 706,855 Norwegian CYP found an increase in primary care use after SARS-COV-2 infection which persisted for up to six months among 1–5-year-olds.^8^

Our study is unique, examining within-individual longitudinal data after laboratory testing for SARS-CoV-2 in test-positive and test-negative CYP, and provides added value over repeated cross-sectional prevalence surveys. Indeed, the two follow-up time points is a major study strength, although more follow-up and continuous time-points would further strengthen the study. This is in-part why we present the sensitivity analysis on the sub-sample with an additional follow-up time point (at 3-months). Notably, we were specifically funded to study non-hospitalised CYP, the milder end of the acute COVID-19 spectrum, which is likely to be relevant to many COVID-19 cases in CYP. However, anecdotal reports from carers and clinical colleagues suggest that there are undoubtedly some CYP severely affected by chronic debilitating long-term symptoms.

The CLoCk study has limitations which have been discussed at length^11^^,^^12^ and here we detail main limitations relevant to the current manuscript. Symptoms at baseline are subject to recall bias as they were reported at time of first contact with the CLoCk study (at either 3-months or 6-months post-test); however, 6-month and 12-month symptoms were reported prospectively. The dominant UK virus was the original wild-type SARS-COV-2 between September and December 2020 and the Alpha (B.1.1.7) variant from January to March 2021; our cohort was drawn from these two periods. From June 2021 the Delta variant dominated and from January 2022, Omicron. In relation to symptoms at the time of the acute infection, evidence suggests that the seven most prevalent symptoms were common to both Alpha and Delta variants.^24^ However, given we excluded test-positives who were reinfected and test-negatives who were infected after baseline testing (PCR testing remained widely available in the UK throughout the 12-month follow-up period and we also took into consideration self-report of Lateral flow tests), our study did not include CYP infected with Delta or Omicron variants and cannot therefore be definitive about post-COVID-19 condition in CYP infected with Delta or Omicron variants. Moreover, it is possible that some CYP might have been misdiagnosed as SARS-CoV-2 negative and vice-versa: false negatives might be attributable to the timing of the PCR, swab technique, and assay sensitivity, but false-positive PCR results are rare. The response rate for the 6-month follow-up questionnaire was 11.2% (14,384 of 127,896; Fig. 1) and at 12 months 48.7% (6307 of 12,949; Fig. 1), but there was little difference in demographic characteristics between respondents and the target population, nor between test-positive and test-negative participants (with the exception of age; Table 1), reflecting the matched-cohort study design. However, we note that the study design may induce selection biases, for example, by favouring those with internet access, and CYP may be more likely to take participate if they had symptoms to report. We acknowledge the limitations of examining self-reported data, compared to in-person medical interviews which were not practical or feasible to conduct. However, we also note that self-report is an appropriate data collection technique for large scale epidemiological studies such as CLoCk. Our unique study emphasises the importance of longitudinal follow-up in the same individuals over time alongside matched test-negatives to avoid the pitfalls of repeated cross-sectional prevalence studies. Whilst we have examined adverse health and well-being at 6- and 12-months post-test (and in a subsample at 3-months post-test), we cannot infer whether these adverse symptoms waxed and waned in the intervening time-periods. While the research definition of ‘Long Covid’ in CYP^22^ rightly requires that the experienced symptoms have an impact on everyday functioning, it is our view that understanding the impact of individual symptoms as well as their collective impact is required to fully understand the impairment resulting from SARS-COV-2 infection. Therefore, in this paper we report the prevalence of symptoms which were assessed by single items as well as reporting validated scales and our operationalisation of the research definition of Long COVID. Nonetheless, we acknowledge that some symptoms (e.g., shortness of breath) might be better assessed by additional validated measures and acknowledge the issue of floor/ceiling effects (i.e., if the question/validated scale is relatively easy or difficult such that substantial proportions of CYP obtain either maximum or minimum scores and the true extent of their abilities cannot be determined). In relation to the data collected, researchers want to ask about as much as possible to allow extensive/varied analysis addressing as many specific research questions as possible and our initial draft questionnaire took over an hour to complete. However, in our pilot study CYP said they would only be willing to spend 20 minutes maximum completing the survey. Therefore, compromises were made and while our data is wide ranging and unique, adding value to the literature, it also has limitations in terms of depth of information available. Finally, much remains unknown in relation to the long-term implications of SARS-COV-2 infection in CYP and as the background epidemiological situation in relation to SARS-COV-2 infection prevalence changes, as well as the rate of vaccination up-take in CYP, more questions need answering, such as, how does vaccination status influences subsequent outcomes after SARS-COV-2 infection?

### Conclusions

In CYP, the prevalence of adverse health and well-being reported at the time of a positive PCR-test declined over 12 months. New adverse symptoms were reported 6- and 12-months post-test for both test-positives and test-negatives, particularly tiredness, shortness of breath, poor quality of life, having emotional and behavioural difficulties, poor well-being, fatigue and Long COVID (according to the Delphi definition).^22^ Such common symptoms may be caused by multiple factors including SARS-COV-2 infection in CYP.

## Contributors

Terence Stephenson t.stephenson@ucl.ac.uk conceived the idea for the study, submitted the successful grant application and drafted the manuscript.

Snehal M Pinto Pereira snehal.pereira@ucl.ac.uk designed and conducted the statistical analyses for the manuscript, accessed and verified the data and drafted the manuscript.

Roz Shafran r.shafran@ucl.ac.uk contributed to the design of the study, submitted the ethics and R&D applications and drafted the manuscript.

Manjula D Nugawela manjula.nugawela@ucl.ac.uk conducted the statistical analysis for the manuscript, accessed and verified the data.

Kelsey McOwat Kelsey.Mcowat@ukhsa.gov.uk adapted the questionnaire for the online SNAP survey platform.

Ruth Simmons Ruth.Simmons@ukhsa.gov.uk accessed and verified the data, designed the participant sampling and dataflow.

Trudie Chalder trudie.chalder@kcl.ac.uk contributed to the design of the study and reviewed the manuscript.

Tamsin Ford tjf52@medschl.cam.ac.uk contributed to the design of the study and reviewed the manuscript.

Isobel Heyman i.heyman@ucl.ac.uk contributed to the design of the study reviewed the manuscript.

Shamez Ladhani shamez.ladhani@ukhsa.gov.uk developed the study methodology, operationalised the regulatory and recruitment ideas for the study and revised the manuscript.

Emma Dalrymple e.dalrymple@ucl.ac.uk contributed to the design of the study and reviewed the manuscript.

Dougal Hargreaves d.hargreaves@imperial.ac.uk contributed to the design of the study, the drafting and the analysis of school attendance data, and reviewed the manuscript.

Simon R White sw539@medschl.cam.ac.uk contributed to the analysis and reviewed the manuscript.

Laura Panagi lp579@medschl.cam.ac.uk contributed to the drafting, analysis and reviewed the manuscript.

Kishan Sharma kishan.sharma@mft.nhs.uk contributed to the design of the study.

Natalia K Rojas n.rojas@ucl.ac.uk contributed to the analysis and reviewed the manuscript.

Sophie D Bennett sophie.bennett.10@ucl.ac.uk reviewed the manuscript.

All members of the CLoCk Consortium (listed below) made contributions to the conception or design of the study; and were involved in drafting the original funding application. All authors of this manuscript; approved the version to be published; and agree to be accountable for all aspects of the work in ensuring that questions related to the accuracy or integrity of any part of the work are appropriately investigated and resolved.

**Additional Co-Applicants on the grant application and CLoCk Consortium members (alphabetical)**

Marta Buszewicz, University College London, m.buszewicz@ucl.ac.uk.

Esther Crawley, University of Bristol, Esther.Crawley@bristol.ac.uk.

Bianca De Stavola, University College London, b.destavola@ucl.ac.uk.

Shruti Garg, University of Manchester, Shruti.Garg@mft.nhs.uk.

Anthony Harnden, Oxford University, anthony.harnden@phc.ox.ac.uk.

Michael Levin, Imperial College London, m.levin@imperial.ac.uk.

Vanessa Poustie, University of Liverpool, v.poustie@liverpool.ac.uk.

Terry Segal, University College London Hospitals NHS Foundation Trust, terry.segal@nhs.net.

Malcolm Semple, University of Liverpool, M.G.Semple@liverpool.ac.uk.

Olivia Swann, Edinburgh University, Olivia.Swann@ed.ac.uk.

Elizabeth Whittaker, Imperial College London, e.whittaker@imperial.ac.uk.

## Data sharing statement

Data is not publicly available. All requests for data will be reviewed by the Children & young people with Long Covid (CLoCk) study team, to verify whether the request is subject to any intellectual property or confidentiality obligations. Requests for access to the participant-level data from this study can be submitted via email to Clock@phe.gov.uk with detailed proposals for approval. A signed data access agreement with the CLoCk team is required before accessing shared data. Code is not made available as we have not used custom code or algorithms central to our conclusions.

## Declaration of interests

Terence Stephenson is Chair of the 10.13039/100005622Health Research Authority and therefore recused himself from the Research Ethics Application. Trudie Chalder is a member of the National Institute for Health and Care Excellence committee for long COVID. She has written self-help books on chronic fatigue and has done workshops on chronic fatigue and post infectious syndromes. Dougal Hargreaves had a part-time secondment as Deputy Chief Scientific Adviser from September 2020 to September 2021, whereby his salary for 2 days per week was paid by the Department for Education (England) to Imperial College London. Sophie Bennett and Roz Shafran are both part of Great Ormond Street Hospital 10.13039/100015819NHS Foundation Trust and UCL 10.13039/501100001282Great Ormond Street Institute of Child Health, where their research is made possible by the National Institute of Health Research (NIHR) 10.13039/501100019256Great Ormond Street Hospital Biomedical Research Centre. Sophie Bennett and Roz Shafran are co-authors on a book published in August 2020, titled *Oxford Guide to Brief and Low Intensity Interventions for Children and Young People*.

All remaining authors have no conflicts of interest.

## Appendix A Supplementary data

Supplementary Material Revision 1

LANCET STROBE
