# Supplementary material for: Natural course of health and well-being in non-hospitalised children and young people after testing for SARS-CoV-2: a prospective follow-up study over 12 months
Source: Lancet Reg Health Eur. 2022 Dec 5;25:100554. doi: 10.1016/j.lanepe.2022.100554 (PMC9719829; doi:10.1016/j.lanepe.2022.100554)
Supplement: Supplementary Material Revision 1 [file mmc1.docx]

**Table of Contents**

| **Content** | **Page(s)** |
| --- | --- |
| Supplementary Text A | 2 |
| Supplementary Text B | 2 |
| Supplementary Table 1 | 3 |
| Supplementary Table 2 | 4-5 |
| Supplementary Table 3 | 6 |
| Supplementary Table 4 | 7-8 |
| Supplementary Table 5 | 9 |
| Supplementary Figure 1 | 10 |
| Supplementary Figure 2 | 11 |
| Supplementary Figure 3 | 12 |
| Supplementary Figure 4 | 13 |
| Supplementary Figure 5 | 14 |
| Supplementary Figure 6 | 15 |
| Supplementary Figure 7 | 16 |
| Supplementary Figure 8 | 17 |
| Supplementary Figure 9 | 18 |
| Supplementary Figure 10 | 19 |
| Supplementary Figure 11 | 20 |
| Supplementary Figure 12 | 21 |
| Supplementary Figure 13 | 22-23 |
| References | 24 |

**Supplementary Text A**

Minor changes were made to the survey between the 6- and 12-month follow-up. These changes included the addition of a symptom severity scale, a symptom impact scale and additional questions on the type of COVID-19 vaccination received and date each dose was administered.

**Supplementary Text B: CYPs’ school attendance 6 months after confirmed COVID-19 infection: exploratory analysis**

While more evidence is now available on the effects of SARS-CoV-2 infection in CYP^1,2^ little is known about the long-term impact on school attendance. This information is needed to guide education support strategies, as well as informing decisions about potential benefits and costs of future public health measures. Thus, we explored self-reported school absence data in CLoCk participants 6 months after initial PCR-testing. We used chi squared tests and logistic regression models to compare recent school absences between CYP who SARS-CoV-2 PCR were positive versus PCR-negative (Supplementary Figure 13).

Among symptomatic CYP, school absence (≥1 day) at 6 months was *less* common in SARS-CoV-2 PCR-positive participants than PCR-negative participants (624/2306 [27.1%] vs 220/540 [40.7%], p<0.001), but a higher proportion reported *extended school absence* of >10 days (288/2306 [12.5%] vs. 21/540 [3.9%], p<0.001) (Supplementary Table 5). Among symptomatic SARS-CoV-2 PCR-positive adolescents, prolonged school absence at 6 months was more common in those who reported ≥3 symptoms at the time of PCR-testing than those reporting 1-2 symptoms (253/1853 [13.7%] vs. 35/453 [7.7%], p<0.001). After adjusting for age, gender, ethnicity, Index of Multiple Deprivation, prior physical health and mental health status, symptomatic PCR-positive CYP had lower odds of any recent school absence (vs. no absence) when compared to symptomatic PCR-negative CYP (aOR 0.56; 95% CI, 0.46-0.68, p<0.001), but higher odds of extended school absence (aOR 3.90; 95%CI, 2.46-6.16; p<0.001) compared to ≤10 days absence.

Compared to symptomatic CYP, adolescents who were asymptomatic reported lower absence rates 6 months after testing. There were also no significant differences between PCR-positive vs. PCR-negative asymptomatic CYP in terms of absences at 6 months post-test. However, due to the study methodology, the symptomatic and asymptomatic groups are not directly comparable, and the asymptomatic group could be underpowered to detect meaningful attendance differences (Supplementary Figure 13).

A strength of this exploration is the ability to recruit SARS-CoV-2 infected and non-infected CYP through a national testing database, removing CYP with re-infection among test-positives and subsequent infections among test-negatives from the analysis, and obtaining detailed follow-up data from online questionnaires. In addition to the limitations of the overall study design, which have been described in the main text, another important limitation is that the questionnaire period included part of the summer holidays. As a result, absence rates are lower than if the full study period had been during term time and thus direct comparison with published term-time absence rates are not possible. While we excluded participants who tested positive (PCR or self-report) following their first PCR test, it is likely that some participants were absent from school due to isolation requirements. In March 2021, 10 days isolation was advised for all close contacts of a case.^3^ The rules changed over time so that by September 2021, isolation was not required for asymptomatic contacts and the duration of isolation was decreased for some groups.^4,5^ We defined extended absence as greater than 10 days to exclude those whose only absence was for a single period of isolation.

To our knowledge, this is the first national study to investigate the association between SARS-CoV-2 infection among 11–17-year-olds and their school attendance 6 months later. Among symptomatic SARS-CoV-2 PCR-positive CYP, extended school absence (>10 days) at 6 months was more common than in PCR-negative adolescents. The reverse pattern was seen for any school absence, which was more common in PCR-negative adolescents. Further studies should assess the impact of persistent symptoms alongside other potential causal factors on education and school attendance in adolescents following COVID-19 infection.

**Supplementary Table 1.** Information on measures included in the CLoCk questionnaire and details on how they have been dichotomised

| Measure | Details (including how measures have been dichotomised) | Time points of data collection (months)* |
| --- | --- | --- |
| 21 symptoms | Mostly assessed as present/absent^6^ | 0, 3, 6, 12 |
| Quality of life/functioning (EQ-5D-Y) | For mobility, looking after self, doing usual activities, and having pain or discomfort: experiencing some or a lot of problems; for worried/sad/unhappy: feeling very worried, sad or unhappy.^7^ | 0, 3, 6, 12 |
| Loneliness (UCLA Loneliness Scale) | 3 items from the UCLA Loneliness Scale were summed to create a total score (range: 3-9); lonely defined as a total score ≥8.^8,9^ | 0, 3, 6, 12 |
| Strengths and Difficulties Questionnaire (SDQ) | 25 items that are combined to form five subscales (five items each): emotional symptoms, conduct problems, hyperactivity, peer relationships and the prosocial skills subscale. Each of the 25 items are scored from 0 to 2, giving a score for each subscale ranging from 0 to 10. All the subscales, except the prosocial subscale, are summed to produce a total difficulties score ranging from 0 to 40. A further impact subscale score indicates the impact of difficulties on CYP in terms of distress and social impairment; it is scored 0-10 with increasing impact producing a higher score.  We used established cut-off points: ≥18 (total difficulties); ≥6 (emotional symptoms), ≥5 (conduct problems), ≥7 (hyperactivity); ≥4 (peer difficulties); ≤5 (prosocial skills) and ≥2 for impact.^10^ | 3, 6, 12 |
| Short Warwick Edinburgh Mental Wellbeing Scale (SWEMWS) | Responses to 7-items provided using a 5-point scale (scored from 1 to 5). Summed scores provide a mental wellbeing score (range 7-35). We converted to metric and used the established cut-off point of <19.6^11^ to reflect poor wellbeing. | 3, 6, 12 |
| Fatigue (Chalder Fatigue Scale; CFQ-11) | 11 items were each dichotomised (4-point scale and coded as: 0,0,1,1). The 11 dichotomised variables were summed to a 0-11 scale. Fatigue was defined using the established cut-off of ≥ 4.^12,13^ | 3, 6, 12 |
| Long COVID | Using data from the questionnaire on the 21 symptoms and the EQ-5D-Y scale (see details above), the Delphi research definition of long COVID^14^ was operationalized as having at least 1 symptom and experiencing some/a lot of problems with respect to mobility, self-care, doing usual activities or having pain/discomfort or feeling very worried/sad. The need for a positive test result was not required when assessing how many test-negatives would also have met this definition. | 3, 6, 12 |

*all 0 month data collection was retrospective; 3-month data collection available on only a sub-sample (see Methods for details)

**Supplementary Table 2.** Prevalence N (%) of CYP reporting the same adverse measures at testing, at 6 months post-test, at 12 months post-test (never, once, twice or at all three time points)

|  | SARS-CoV-2 PCR-Negative (N=2177) | | | | SARS-CoV-2 PCR-Positive (N=2909) | | | |  |
| --- | --- | --- | --- | --- | --- | --- | --- | --- | --- |
|  | **Never had adverse measure** | **Only once** | **Twice** | **Three times** | **Never had adverse measure** | **Only once** | **Twice** | **Three times** | **p-value*** |
| Symptom |  |  |  |  |  |  |  |  |  |
| Fever | 2017(92·7%) | 157(7·2%) | 3(0·1%) | 0(0·0%) | 2224(76·5%) | 653(22·4%) | 31(1·1%) | 1(0·0%) | <0·001 |
| Chills or shivers | 1852(85·1%) | 268(12·3%) | 54(2·5%) | 3(0·1%) | 2028(69·7%) | 694(23·9%) | 167(5·7%) | 20(0·7%) | <0·001 |
| Persistent cough | 1912(87·8%) | 236(10·8%) | 29(1·3%) | 0(0·0%) | 2249(77·3%) | 568(19·5%) | 87(3·0%) | 5(0·2%) | <0·001 |
| Unusual Fatigue/tiredness | 1234(56·7%) | 557(25·6%) | 360(16·5%) | 26(1·2%) | 1039(35·7%) | 806(27·7%) | 748(25·7%) | 316(10·9%) | <0·001 |
| Unusual shortness of breath | 1754(80·6%) | 296(13·6%) | 120(5·5%) | 7(0·3%) | 1817(62·5%) | 586(20·1%) | 379(13%) | 127(4·4%) | <0·001 |
| Loss of smell/taste | 2086(95·8%) | 78(3·6%) | 9(0·4%) | 4(0·2%) | 1860(63·9%) | 738(25·4%) | 216(7·4%) | 95(3·3%) | <0·001 |
| Unusually hoarse voice | 2069(95·0%) | 97(4·5%) | 11(0·5%) | 0(0·0%) | 2686(92·3%) | 201(6·9%) | 20(0·7%) | 2(0·1%) | 0·001 |
| Unusual chest pain | 1988(91·3%) | 152(7·0%) | 37(1·7%) | 0(0·0%) | 2368(81·4%) | 418(14·4%) | 106(3·6%) | 17(0·6%) | <0·001 |
| Unusual abdominal pain | 2037(93·6%) | 120(5·5%) | 17(0·8%) | 3(0·1%) | 2567(88·2%) | 284(9·8%) | 50(1·7%) | 8(0·3%) | <0·001 |
| Diarrhoea | 2073(95·2%) | 88(4·0%) | 15(0·7%) | 1(0·0%) | 2657(91·3%) | 215(7·4%) | 28(1·0%) | 9(0·3%) | <0·001 |
| Headache | 1686(77·4%) | 362(16·6%) | 124(5·7%) | 5(0·2%) | 1507(51·8%) | 954(32·8%) | 347(11·9%) | 101(3·5%) | <0·001 |
| Confusion, disorientation, or drowsiness | 2036(93·5%) | 117(5·4%) | 22(1·0%) | 2(0·1%) | 2466(84·8%) | 344(11·8%) | 85(2·9%) | 14(0·5%) | <0·001 |
| Unusual eye-soreness or discomfort | 2013(92·5%) | 143(6·6%) | 20(0·9%) | 1(0·0%) | 2432(83·6%) | 396(13·6%) | 69(2·4%) | 12(0·4%) | <0·001 |
| Skipping meals | 1913(87·9%) | 216(9·9%) | 45(2·1%) | 3(0·1%) | 2212(76%) | 502(17·3%) | 164(5·6%) | 31(1·1%) | <0·001 |
| Dizziness or light-headedness | 1868(85·8%) | 238(10·9%) | 67(3·1%) | 4(0·2%) | 2010(69·1%) | 612(21%) | 238(8·2%) | 49(1·7%) | <0·001 |
| Sore throat | 1828(84·0%) | 299(13·7%) | 45(2·1%) | 5(0·2%) | 1952(67·1%) | 824(28·3%) | 117(4·0%) | 16(0·6%) | <0·001 |
| Unusual strong muscle pains | 2048(94·1%) | 118(5·4%) | 10(0·5%) | 1(0·0%) | 2406(82·7%) | 416(14·3%) | 74(2·5%) | 13(0·4%) | <0·001 |
| Earache or ringing in your ears | 1984(91·1%) | 147(6·8%) | 45(2·1%) | 1(0·0%) | 2483(85·4%) | 318(10·9%) | 90(3·1%) | 18(0·6%) | <0·001 |
| Raised, red, itchy welts on the skin | 2140(98·3%) | 34(1·6%) | 2(0·1%) | 1(0·0%) | 2813(96·7%) | 83(2·9%) | 11(0·4%) | 2(0·1%) | 0·004 |
| Red/purple sores or blisters on feet | 2143(98·4%) | 29(1·3%) | 5(0·2%) | 0(0·0%) | 2835(97·5%) | 65(2·2%) | 8(0·3%) | 3(0·1%) | 0·064 |
| Other | 1990(91·4%) | 175(8·0%) | 12(0·6%) | 0(0·0%) | 2480(85·3%) | 381(13·1%) | 46(1·6%) | 2(0·1%) | <0·001 |
| Quality of life/functioning  (EQ-5D-Y)** |  |  |  |  |  |  |  |  |  |
| Mobility | 1925 (88.4%) | 149 (6.8%) | 57 (2.6%) | 46 (2.2%) | 2579 (88.8%) | 222 (7.6%) | 76 (2.6%) | 30 (1.0%) | 0.013 |
| Self-care | 1992 (91.5%) | 90 (4.1%) | 56 (2.6%) | 39 (1.8%) | 2719 (93.5%) | 121 (4.2%) | 45 (1.6%) | 22 (0.7%) | <0.001 |
| Pain | 1527 (70.1%) | 359 (16.5%) | 151 (7.0%) | 140 (6.4%) | 1965 (67.6%) | 535 (18.4%) | 275 (9.5%) | 132 (4.5%) | <0.001 |
| Usual activities | 1689 (77.6%) | 284 (13.1%) | 127 (5.8%) | 77 (3.5%) | 2144 (73.7%) | 477 (16.4%) | 214 (7.4%) | 72 (2.5%) | <0.001 |
| Feeling sad | 1863 (85.6%) | 178 (8.2%) | 101 (4.6%) | 35 (1.6%) | 2485 (85.5%) | 287 (9.9%) | 101 (3.5%) | 34 (1.1%) | 0.019 |
| UCLA Loneliness Scale |  |  |  |  |  |  |  |  |  |
| Lonely | 1829(84.0%) | 193 (8.9%) | 112 (5.1%) | 43 (1.2%) | 2510 (86.3%) | 259(8.9%) | 98(3.4%) | 42(1.4%) | 0.006 |
| Mental health and well-being*** |  |  |  |  |  |  |  |  |  |
| SDQ |  |  |  |  |  |  |  |  |  |
| High/very high total difficulties | 1620 (74·4%) | 273 (12·5%) | 284 (13·1%) |  | 2211 (76·0%) | 376 (12·9%) | 322 (11·1%) |  | 0·098 |
| High/very high emotional symptoms | 1372 (63·0%) | 386 (17·7%) | 419 (19·3%) |  | 1881 (64·7%) | 515 (17·7%) | 513 (17·6%) |  | 0·318 |
| High/very high conduct problems | 1968 (90·4%) | 143 (6·57%) | 66 (3·03%) |  | 2685 (92·3%) | 162 (5·57%) | 62 (2·13%) |  | 0·037 |
| High/very high hyperactivity | 1627 (74·7%) | 292 (13·4%) | 258 (11·9%) |  | 2185 (75·1%) | 391 (13·4%) | 333 (11·5%) |  | 0·905 |
| High/very high peer difficulties | 1433 (65·8%) | 411 (18·9%) | 333 (15·3%) |  | 2086 (71·7%) | 466 (16·0%) | 357 (12·3%) |  | 0·000 |
| Low/very low prosocial skills | 1727 (79·3%) | 283 (13·0%) | 167 (7·7%) |  | 2372 (81·5%) | 358 (12·3%) | 179 (6·2%) |  | 0·066 |
| High/very high impact** | 1467 (67·4%) | 342 (15·7%) | 321 (14·8 %) |  | 2010 (69·1%) | 489 (16·8%) | 356 (12·2%) |  | 0·026 |
| SWEMWBS |  |  |  |  |  |  |  |  |  |
| Poor wellbeing | 1051 (48·3%) | 564 (25·9%) | 562 (25·8%) |  | 1507 (51·8%) | 747 (25·7%) | 655 (22·5%) |  | 0·012 |
| Severe fatigue*** | 1188 (54·6%) | 516 (23·7%) | 473 (21·7%) |  | 1516 (52·1%) | 636 (21·9%) | 757 (26·0%) |  | 0·002 |
| Long COVID*** | 1569 (72·1%) | 396 (18·2%) | 212 (9·7%) |  | 1841 (63·3%) | 603 (20·7%) | 465 (16·0%) |  | <0·001 |

*p-value from chi-squared test; **N=5,084 (EQ-5D-Y); 4,985 (high/very high impact)

| Supplementary Table 3. Comparison of target population who responded at 3-months to analytic sample; and characteristics of CYP in analytic sample by PCR test result for SARS-CoV-2: N (%) | | | | | |
| --- | --- | --- | --- | --- | --- |
| Characteristic | | **Target population of CYP who responded at 3 months post-testing** | **CYP in analytic sample (responding at 3- 6- and 12-months post-test)** | **PCR test result**  **Negative** | **PCR test result**  **Positive** |
| N |  | 7,238 | 1,808 | 800 | 1,008 |
| PCR test result |  |  |  |  |  |
|  | Negative | 3,943 (54.5) | 800 (44.3) | 800 (44.3) | - |
|  | Positive | 3,295 (45.5) | 1,008 (55.8) | - | 1,008 (55.8) |
| Age (years) |  |  |  |  |  |
|  | 11-14 | 3,075 (42.5) | 712 (39.4) | 304 (38.0) | 408 (40.5) |
|  | 15-17 | 4,163 (57.5) | 1,096 (60.6) | 496 (62.0) | 600 (59.5) |
| Sex |  |  |  |  |  |
|  | Male | 2,686 (37.1) | 622 (34.4) | 299 (37.4) | 323 (32.0) |
|  | Female | 4,552 (62.9) | 1,186 (65.6) | 501 (62.6) | 685 (68.0) |
| Ethnicity |  |  |  |  |  |
|  | White | 5,347 (73.9) | 1,348 (76.6) | 609 (76.1) | 739 (73.3) |
|  | Asian/Asian British | 1,084 (15.0) | 275 (15.2) | 120 (15.0) | 155 (15.4) |
|  | Mixed | 363 (5.0) | 89 (4.9) | 36 (4.5) | 53 (5.3) |
|  | Black/African/Caribbean British | 272 (3.8) | 58 (3.2) | 22 (2.8) | 36 (3.8) |
|  | Other | 119 (1.6) | 27 (1.5) | 9 (1.1) | 18 (1.8) |
|  | Prefer not to say | 53 (0.7) | 11 (0.6) | 4 (0.5) | 7 (0.7) |
| IMD^a^ |  |  |  |  |  |
|  | 1 (most deprived) | 1,484 (20.5) | 304 (16.8) | 133 (16.6) | 171 (17.0) |
|  | 2 | 1,444 (20.0) | 376 (20.8) | 172 (21.5) | 204 (20.2) |
|  | 3 | 1,385 (19.1) | 363 (20.1) | 168 (21.0) | 195 (19.4) |
|  | 4 | 1,400 (19.3) | 362 (20.0) | 159 (19.9) | 203 (20.1) |
|  | 5 (least deprived) | 1,525 (21.1) | 403 (22.3) | 168 (21.0) | 235 (23.3) |
| Region |  |  |  |  |  |
|  | East Midlands | 674 (9.3) | 168 (9.3) | 78 (9.8) | 90 (8.9) |
|  | East of England | 1,153 (15.3) | 296 (16.4) | 132 (16.5) | 164 (16.3) |
|  | London | 1,207 (16.7) | 293 (16.2) | 141 (17.6) | 152 (15.1) |
|  | North East | 242 (3.3) | 51 (2.8) | 21 (2.6) | 30 (3.0) |
|  | North West | 850 (11.7) | 192 (10.6) | 80 (10.0) | 112 (11.1) |
|  | South East | 1,193 (16.5) | 332 (18.4) | 145 (18.1) | 187 (18.6) |
|  | South West | 555 (7.7) | 152 (8.4) | 65 (8.1) | 87 (8.6) |
|  | West Midlands | 868 (12.0) | 223 (12.3) | 97 (12.1) | 126 (12.5) |
|  | Yorkshire and The Humber | 496 (6.9) | 101 (5.6) | 41 (5.1) | 60 (6.0) |
| ^a^Index of Multiple Deprivation, derived from the children and young people’s lower super output area (a small local area level based geographic hierarchy), was used as a proxy for socio-economic status. We used Index of Multiple Deprivation quintiles from most (quintile 1) to least (quintile 5) deprived. | | | | | |

**Supplementary Table 4.** Prevalence N (%) of CYP reporting the same adverse measure at testing, at 3 months post-test, 6 months post-test and at 12 months post-test (never, once, twice, trice or at all four time points)

|  | SARS-CoV-2 PCR-Negative (N=800) | | | |  | SARS-CoV-2 PCR-Positive (N=1,008) | | | |  |  |
| --- | --- | --- | --- | --- | --- | --- | --- | --- | --- | --- | --- |
|  | **Never had adverse measure** | **Only once** | **Twice** | **Trice** | **Four times** | **Never had adverse measure** | **Only once** | **Twice** | **Trice** | **Four times** | **p-value*** |
| Symptom |  |  |  |  |  |  |  |  |  |  |  |
| Fever | 748 (93.5) | 51 (6.4) | 1 (0.1) | 0 | 0 | 791 (78.5) | 206 (20.4) | 9 (0.9) | 2 (0.2) | 0 | <0.001 |
| Chills or shivers | 678 (84.7) | 91(11.4) | 24 (3.0) | 6 (0.7) | 1 (0.1) | 685 (67.9) | 227 (22.5) | 62 (6.2) | 30 (3.0) | 4 (0.4) | <0.001 |
| Persistent cough | 719 (89.9) | 67 (8.4) | 11 (1.3) | 3 (0.4) | 0 | 784 (77.8) | 191 (18.9) | 26 (2.5) | 6 (0.6) | 1 (0.1) | <0.001 |
| Unusual Fatigue/tiredness | 413 (51.6) | 182 (22.8) | 120 (15.0) | 81 (10.1) | 4 (0.5) | 307 (30.5) | 256 (25.4) | 157 (15.5) | 201 (19.9) | 87 (8.6) | <0.001 |
| Unusual shortness of breath | 633 (79.1) | 100 (12.5) | 35 (4.4) | 31 (3.9) | 1 (0.1) | 594 (58.9) | 175 (17.4) | 107 (10.6) | 99 (9.8) | 33 (3.3) | <0.001 |
| Loss of smell/taste | 763 (95.4) | 31 (3.9) | 3 (0.3) | 1 (0.1) | 2 (0.3) | 682 (67.7) | 211 (20.9) | 58 (5.7) | 38 (3.8) | 19 (1.9) | <0.001 |
| Unusually hoarse voice | 761 (95.1) | 35 (4.4) | 4 (0.5) | 0 | 0 | 918 (91.1) | 79 (7.8) | 6 (0.6) | 5 (0.5) | 0 | 0.004 |
| Unusual chest pain | 718 (89.7) | 64 (8.0) | 14 (1.7) | 4 (0.5) | 0 | 796 (79.0) | 150 (14.8) | 44 (4.4) | 15 (1.5) | 3 (0.3) | <0.001 |
| Unusual abdominal pain | 734 (91.7) | 56 (7.0) | 7 (0.9) | 2 (0.3) | 1 (0.1) | 877 (87.0) | 102 (10.1) | 19 (1.9) | 10 (1.0) | 0 | 0.007 |
| Diarrhoea | 757 (94.6) | 34 (4.3) | 6 (0.7) | 3 (0.4) | 0 | 893 (88.6) | 95 (9.4) | 16 (1.6) | 4 (0.4) | 0 | <0.001 |
| Headache | 564 (70.5) | 164 (20.5) | 53 (6.6) | 16 (2.0) | 3 (0.4) | 460 (45.6) | 306 (30.4) | 135 (13.4) | 83 (8.2) | 24 (2.4) | <0.001 |
| Confusion, disorientation, or drowsiness | 731 (91.4) | 54 (6.7) | 7 (0.9) | 7 (0.9) | 1 (0.1) | 815 (80.9) | 132 (13.1) | 40 (4.0) | 18 (1.7) | 3 (0.3) | <0.001 |
| Unusual eye-soreness or discomfort | 731 (91.4) | 54 (6.7) | 11 (1.4) | 4 (0.5) | 0 | 800 (79.4) | 158 (15.6) | 30 (3.0) | 19 (1.9) | 1 (0.1) | <0.001 |
| Skipping meals | 679 (84.9) | 90 (11.3) | 24 (3.0) | 6 (0.7) | 1 (0.1) | 719 (71.3) | 186 (18.5) | 68 (6.7) | 32 (3.2) | 3 (0.3) | <0.001 |
| Dizziness or light-headedness | 656 (82.0) | 92 (11.5) | 31 (3.9) | 19 (2.4) | 2 (0.2) | 650 (64.5) | 219 (21.7) | 84 (8.3) | 48 (4.7) | 7 (0.7) | <0.001 |
| Sore throat | 660 (82.5) | 119 (14.9) | 14 (1.8) | 7 (0.8) | 0 | 631 (62.6) | 305 (30.3) | 61 (6.0) | 11 (1.1) | 0 | <0.001 |
| Unusual strong muscle pains | 752 (94.0) | 40 (5.0) | 6 (0.8) | 2 (0.2) | 0 | 809 (80.2) | 149 (14.8) | 37 (3.7) | 12 (1.2) | 1 (0.1) | <0.001 |
| Earache or ringing in your ears | 709 (88.6) | 62 (7.8) | 16 (2.0) | 12 (1.5) | 1 (0.1) | 829 (82.2) | 127 (12.7) | 37 (3.6) | 14 (1.4) | 1 (0.1) | 0.002 |
| Raised, red, itchy welts on the skin | 780 (97.5) | 20 (2.5) | 0 | 0 | 0 | 967 (95.9) | 37 (3.7) | 2 (0.2) | 2 (0.2) | 0 | 0.156 |
| Red/purple sores or blisters on feet | 779 (97.4) | 17 (2.1) | 3 (0.4) | 1 (0.1) | 0 | 979 (97.1) | 23 (2.3) | 4 (0.4) | 2 (0.2) | 0 | 0.997 |
| Other | 632 (79.0) | 152 (19.0) | 15 (1.9) | 1 (0.1) | 0 | 809 (80.3) | 173 (17.1) | 21 92.1) | 4 (0.4) | 1 (0.1) | 0.556 |
| Quality of life/functioning (EQ-5D-Y) |  |  |  |  |  |  |  |  |  |  |  |
| Mobility | 697 (87.1) | 60 (7.5) | 20 (2.5) | 9 (1.1) | 14 (1.8) | 860 (85.3) | 92 (9.1) | 26 (2.6) | 18 (1.8) | 12 (1.2) | 0.425 |
| Self-care | 723 (90.4) | 29 (3.6) | 21 (2.6) | 12 (1.5) | 15 (1.9) | 935 (92.7) | 38 (3.8) | 17 (1.7) | 10 (1.0) | 8 (0.8) | 0.125 |
| Pain | 529 (66.1) | 126 (15.8) | 74 (9.2) | 30 (3.7) | 41 (5.1) | 632 (62.7) | 176 (17.4) | 110 (10.9) | 53 (5.3) | 37 (3.7) | 0.125 |
| Usual activities | 600 (75.0) | 104 (13.0) | 45 (5.6) | 28 (3.5) | 23 (2.9) | 706 (70.0) | 153 (15.2) | 81 (8.0) | 52 (5.2) | 16 (1.6) | 0.012 |
| Feeling sad | 376(47.0) | 122(15.2) | 220(27.5) | 52(6.5) | 30(3.7) | 471(46.7) | 177(17.6) | 265(26.3) | 72(7.1) | 23(2.3) | 0.264 |
| UCLA Loneliness Scale |  |  |  |  |  |  |  |  |  |  |  |
| Lonely | 654(81.7) | 71(8.9) | 38(4.8) | 25(3.1) | 12(1.5) | 834(82.7) | 95(9.4) | 51(5.1) | 12(1.2) | 16(1.6) | 0.076 |
| Mental health and well-being** |  |  |  |  |  |  |  |  |  |  |  |
| SDQ |  |  |  |  |  |  |  |  |  |  |  |
| High/very high total difficulties | 556 (69.5) | 91 (11.4) | 68 (8.5) | 85 (10.6) |  | 707 (70.1) | 138 (13.7) | 68 (6.8) | 95 (9.4) |  | 0.223 |
| High/very high emotional symptoms | 460 (57.5) | 116 (14.5) | 96 (12.0) | 128 (16.0) |  | 586 (58.1) | 160 (15.9) | 119 (11.8) | 143 (14.2) |  | 0.665 |
| High/very high conduct problems | 705 (88.1) | 58 (7.3) | 20 (2.5) | 17 (2.1) |  | 892 (88.5) | 67 (6.7) | 32 (3.2) | 17 (1.7) |  | 0.705 |
| High/very high hyperactivity | 562 (70.3) | 92 (11.5) | 74 (9.3) | 72 (9.0) |  | 694 (68.9) | 153 (15.2) | 84 (8.3) | 77 (7.6) |  | 0.111 |
| High/very high peer difficulties | 464 (58.0) | 133 (16.6) | 85 (10.6) | 118 (14.8) |  | 647 (64.2) | 168 (16.7) | 93 (9.2) | 100 (9.9) |  | 0.006 |
| Low/very low prosocial skills | 612 (76.5) | 92 (11.5) | 57 (7.1) | 39 (4.9) |  | 775 (76.9) | 125 (12.4) | 65 (6.5) | 43 (4.3) |  | 0.807 |
| High/very high impact^β^ | 499 (62.4) | 96 (12.0) | 67 (8.4) | 103 (12.9) |  | 644 (63.9) | 159 (15.8) | 94 (9.3) | 83 (8.2) |  | 0.002 |
| SWEMWBS |  |  |  |  |  |  |  |  |  |  |  |
| Poor wellbeing | 351 (43.9) | 154 (19.3) | 118 (14.8) | 177 (22.1) |  | 443 (44.0) | 213 (21.1) | 180 (17.9) | 172 (17.1) |  | 0.025 |
| Severe fatigue** | 414 (51.8) | 156 (19.5) | 114 (14.3) | 116 (14.5) |  | 478 (47.4) | 182 (18.1) | 142 (14.1) | 206 (20.4) |  | 0.012 |
| Long COVID** | 525 (65.6) | 153 (19.1) | 74 (9.3) | 48 (6.0) |  | 570 (56.6) | 200 (19.8) | 128 (12.7) | 110 (10.9) |  | <0.001 |

*p-value from chi-squared test; **not reported at 0-months, see Supplementary Table 1; **^β^**N = 1,745

**Supplementary Table 5.** Self-reported school absence of symptomatic adolescents 6 months post-test, by baseline PCR result and number of symptoms, CLoCk study, England.

|  | SARS-CoV-2 PCR-test status | | | | | |
| --- | --- | --- | --- | --- | --- | --- |
|  | **Positive** | | | **Negative** | | |
|  | **Number of Symptoms at time of PCR test** | | | **Number of Symptoms at time of PCR test** | | |
|  | **1-2** | **≥3** | **Total** | **1-2** | **≥3** | **Total** |
| N | 453 | 1,853 | 2,306 | 153 | 387 | 540 |
|  |  |  |  |  |  |  |
| Days absent within last four weeks, n (%) |  |  |  |  |  |  |
| 0 | 351 (77.48) | 1,331 (71.83) | 1,682 (72.94) | 108 (70.59) | 212 (54.78) | 320 (59.26) |
| 1-10 | 67 (14.79) | 269 (14.52) | 336 (14.57) | 44 (28.76) | 155 (40.05) | 199 (36.85) |
| >10 | 35 (7.73) | 253 (13.65) | 288 (12.49) | 1 (0.65) | 20 (5.17) | 21 (3.89) |

**Supplementary Figure 1.** Symptoms with low overall prevalence (less than 10%) at testing and 6- and 12-months post-test


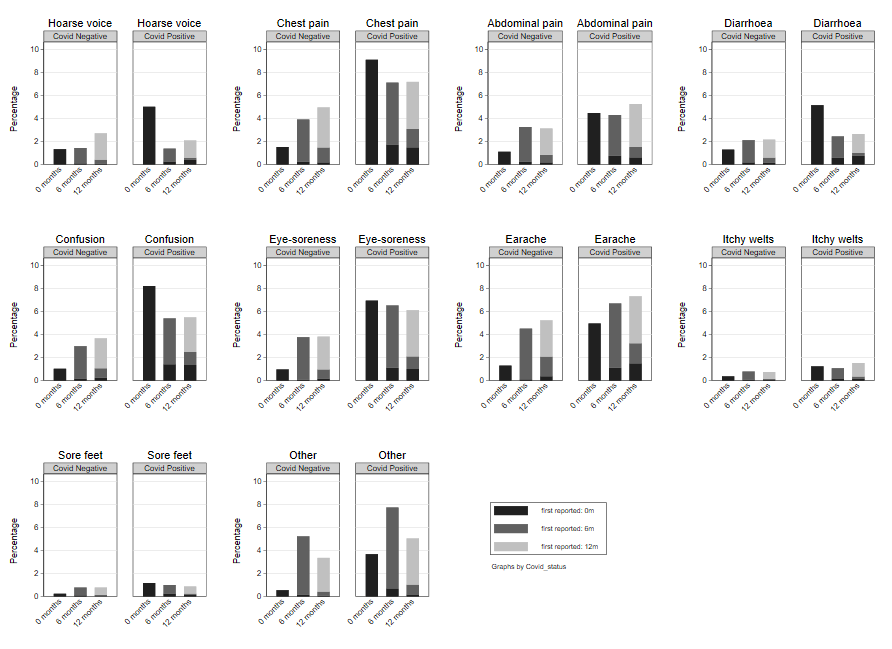


**Supplementary Figure 2.** Symptoms where overall prevalence declined from baseline to 12 months post-test in test-positives*


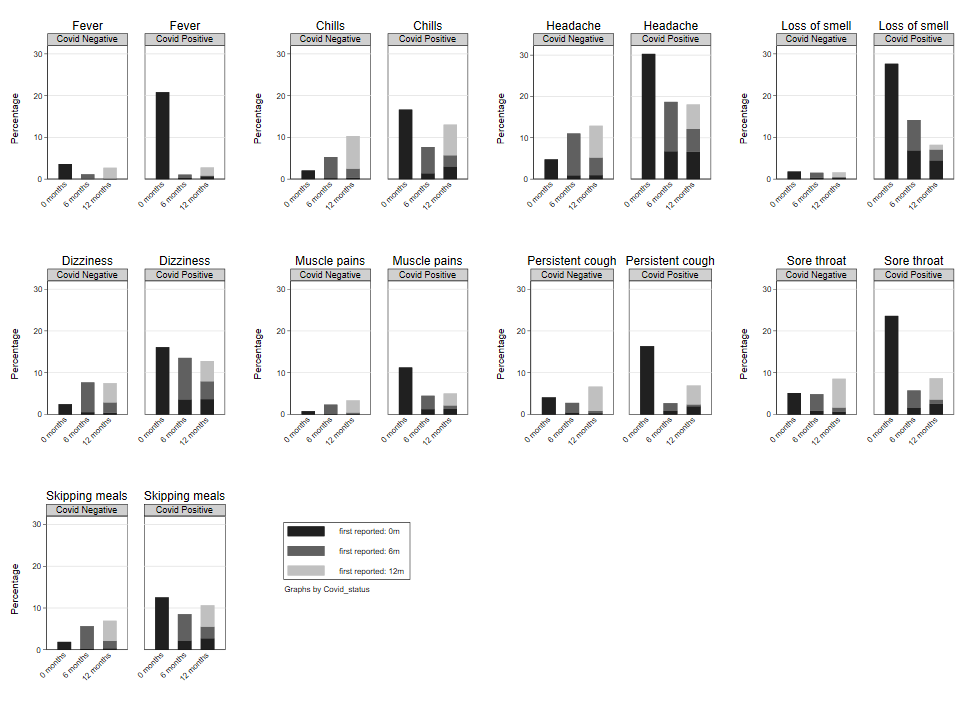


* For all symptoms (except skipping meals) p-value for difference between proportion of CYP with symptom at baseline and 12 months post-test, in test-positives was <0.001; for skipping meals p-value=0.0243.

**Supplementary Figure 3**. Participants’ flow diagram for sub-sample with data collection at 3-months

**Invited to participate at 3 months (N = 50,845)**

- Test-negatives (*n* = 27,797)
- Test-positives (*n* = 23,048)

**Target population: responded at 3m (N = 7,238)**

- Test-negatives (*n* = 3,943)
- Test-positives (*n* = 3,295)

**Excluded late responses** (N = 99)

- Test-negatives (*n* = 50)
- Test-positives (*n* =49)

**Excluded (re)infections*** (N = 16)

- Test-negatives (*n* = 3)
- Test-positives (*n* = 13)

**Responded at 6m (N = 3,851)**

- Test-negatives (*n* = 2,056)
- Test-positives (*n* = 1,795)

**Excluded late responses** (N = 206)

- Test-negatives (*n* = 129)
- Test-positives (*n* = 77)

**Excluded (re)infections*** (N = 188)

- Test-negatives (*n* = 159)
- Test-positives (*n* = 29)

**Responded at 12m (N = 3,179)**

- Test-negatives (*n* = 1,718)
- Test-positives (*n* = 1,461)

**Analytic sample: Responded at 3m, 6m, and 12m after excluding late responses and reinfections* (N = 1,808)**

- Test-negatives (*n* = 800)
- Test-positives (*n* = 1008)

**Excluded late responses** (N = 68)

- Test-negatives (*n* = 39)
- Test-positives (*n* = 29)

**Excluded (re)infections*** (N = 908)

- Test-negatives (*n* = 685)
- Test-positives (*n* = 223)

*(re)infection status determined by (i) PCR test result data held by UKHSA and (ii) self-report by CYP

Note: The 3-, 6-, and 12-month follow-up questionnaires were returned at a median of 14.9 (interquartile range [IQR]: 13, 19.1), 27. 3 (IQR: 25.9, 28.6), and 53.9 (IQR: 52.1, 55.1) weeks post-testing, respectively.

**Supplementary Figure 4**. Symptoms with overall prevalence increasing from time of testing and remaining high at 12 months post-test, in sub-sample with data collected at 3-months

**Supplementary Figure 5.** Symptoms with low overall prevalence (less than 10%*) at all time points, in sub-sample with data collected at 3-months

*except for “other” at 3-months

**Supplementary Figure 6**. Symptoms with overall prevalence decreasing in test-positives from time of testing to 12 months follow-up, in sub-sample with data collected at 3-months

**Supplementary Figure 7.** Prevalence of poor quality of life and functioning* over a 12-month period in test-positives and test-negatives, in sub-sample with data collected at 3-months


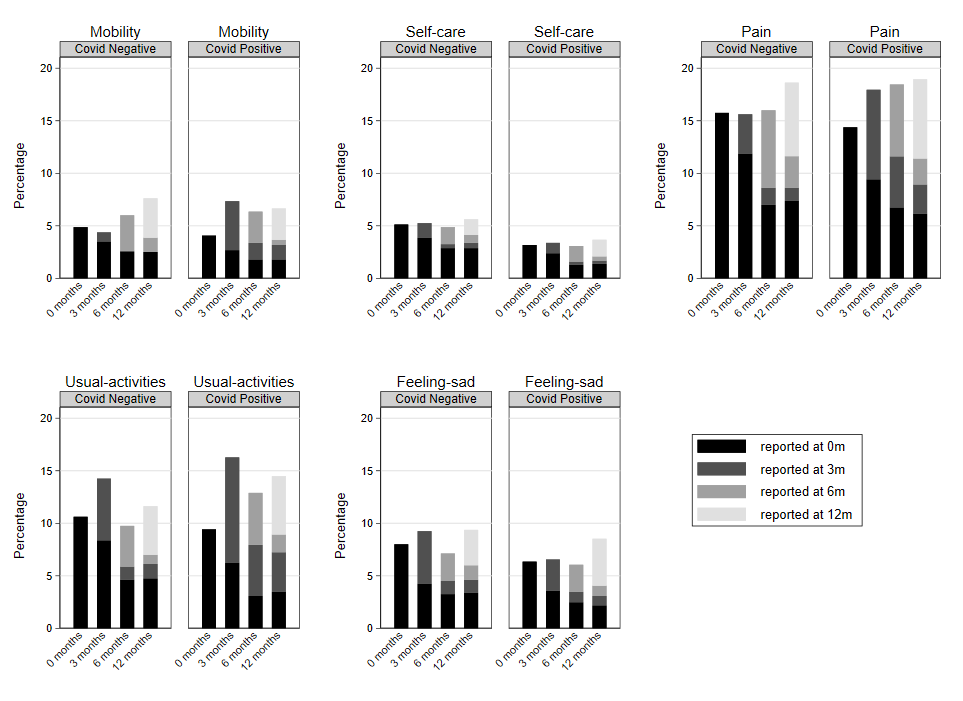


*Questions from the EQ-5D-Y scale, see Supplementary Table 1 for details

**Supplementary Figure 8** Prevalence of loneliness over a 12-month period in test-positives and test-negatives, in sub-sample with data collected at 3-months


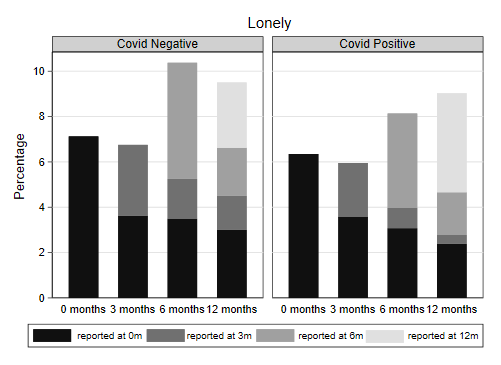


*from UCLA Loneliness scale, see Supplementary Table 1 for details

**Supplementary Figure 9**. Prevalence of emotional and behavioural difficulties* over a 12-month period in test-positives and test-negatives, in sub-sample with data collected at 3-months

*From Strengths and Difficulties Questionnaire, See supplementary Table 1 for details.

**Supplementary Figure 10.** Prevalence of poor well-being* over a 12-month period in test-positives and test-negatives, in sub-sample with data collected at 3-months

Poor well-being

*From the Short Warwick Edinburgh Mental Wellbeing Scale; See supplementary Table 1 for details.

**Supplementary Figure 11**. Prevalence of severe fatigue* over a 12-month period in test-positives and test-negatives, in sub-sample with data collected at 3-months

*From the Chalder Fatigue Scale; see Supplementary Table 1 for details

**Supplementary Figure 12.** Prevalence of Long Covid over a 12-month period in test-positives and test-negatives, in sub-sample with data collected at 3-months

*using data from the Long COVID Delphi definition; see supplementary Table 1 for details (note for test-negatives we excluded the need for a positive test-result)

**Supplementary Figure 13.** Participant flow diagram showing 6-month school attendance data, CLoCK study, England.

Excluded due to missing data on school attendance (N=446):

- Test-negatives (n=81)
- Test-positives (n=365)

**Symptomatic** & included in analysis (N=2,846)

- Test-negatives (n=540)
- Test-positives (n=2,306)

Excluded (N=1,435) due to:

- Responding >34 weeks from time of PCR test (n=1,116)
  - Test-negatives (n=689)
  - Test-positives (n=427)
- Negative at baseline and subsequently infected (n=273)
- Positive at baseline and reinfected (n=46)

Contacted (N=127,896)

- Test-negatives (n=72,449)
- Test-positives (n=55,447)

CYP returning survey (N=14,384)

- Test-negatives (n=7,504)
- Test-positives (n=6,880)

CYP responding within 34 weeks of PCR test and not (re)infected (N=12,949)

- Test-negatives (n=6,542)
- Test-positives (n=6,407)

Symptomatic and responded between 13/04/2021 and 19/10/2021 (N=3,292)

- Test-negatives (n=621)
- Test-positives (n=2,671)

Asymptomatic and responded between 21/08/2021 and 02/11/2021 (N=523)

- Test-negatives (n=270)
- Test-positives (n=253)

**Asymptomatic** & included in analysis (N=523)

- Test-negatives (n=270)
- Test-positives (n=253)

Notes

1. Participants were invited to complete a questionnaire 6 months following their SARS-CoV-2 PCR test. They were asked to report the number of days of school absence over the previous 4 weeks. Responses completed more than 34 weeks after PCR test were not included in the analysis.
2. The study protocol was amended during the study to include school attendance questions for participants who were asymptomatic at the time of PCR testing. The reporting period therefore differs between the asymptomatic and symptomatic group.

**References**

1. Magnusson K, Skyrud KD, Suren P, et al. Healthcare use in 700 000 children and adolescents for six months after covid-19: before and after register based cohort study

. *BMJ* 2022; **376**: e066809.

2. Kikkenborg Berg S, Dam Nielsen S, Nygaard U, et al. Long COVID symptoms in SARS-CoV-2-positive adolescents and matched controls (LongCOVIDKidsDK): a national, cross-sectional study. *The Lancet Child & Adolescent Health* 2022; **6**(4): 240-8.

3. Public Health England. COVID-19 Resource Pack for Educational Settings in Yorkshire and the Humber. 2021. <https://www.rotherham.gov.uk/downloads/file/2101/covid-19-early-years-resource-pack> (accessed September 26, 2022).

4. NHS Digital. PHE SW HPT: Guidance for Childcare and Educational Settings in the Management of COVID-19. 2022. <https://www.england.nhs.uk/south/wp-content/uploads/sites/6/2021/08/schools-flowchart-v15-aug-21.pdf> (accessed September 26, 2022).

5. Department for Education. Pupils only need to isolate if they have symptoms of Covid-19 or if they test positive. 2021. <https://educationhub.blog.gov.uk/2021/09/08/pupils-only-need-to-isolate-if-they-have-symptoms-of-covid-19-or-if-they-test-positive/> (accessed September 26, 2022).

6. Stephenson T, Pinto Pereira SM, Shafran R, et al. Physical and mental health 3 months after SARS-CoV-2 infection (long COVID) among adolescents in England (CLoCk): a national matched cohort study. *The Lancet Child & Adolescent Health* 2022; **6**(4): 230-9.

7. Wille N, Badia X, Bonsel G, et al. Development of the EQ-5D-Y: a child-friendly version of the EQ-5D. *Qual Life Res* 2010; **19**(6): 875-86.

8. Office for National Statistics. Measuring loneliness: guidance for use of the national indicators on surveys. 2018. <https://www.ons.gov.uk/peoplepopulationandcommunity/wellbeing/methodologies/measuringlonelinessguidanceforuseofthenationalindicatorsonsurveys#recommended-measures-for-children> (accessed September 26, 2022).

9. Klein EM, Zenger M, Tibubos AN, et al. Loneliness and its relation to mental health in the general population: Validation and norm values of a brief measure. *Journal of Affective Disorders Reports* 2021; **4**: 100120.

10. youthinmind. Information for researchers and professionals about the Strengths & Difficulties Questionnaires. 2022. <https://www.sdqinfo.org/> (accessed September 26, 2022).

11. Ng Fat L, Scholes S, Boniface S, Mindell J, Stewart-Brown S. Evaluating and establishing national norms for mental wellbeing using the short Warwick-Edinburgh Mental Well-being Scale (SWEMWBS): findings from the Health Survey for England. *Qual Life Res* 2017; **26**(5): 1129-44.

12. Chalder T, Berelowitz G, Pawlikowska T, et al. Development of a fatigue scale. *J Psychosom Res* 1993; **37**(2): 147-53.

13. Loge JH, Ekeberg O, Kaasa S. Fatigue in the general Norwegian population: normative data and associations. *J Psychosom Res* 1998; **45**(1): 53-65.

14. Stephenson T, Allin B, Nugawela MD, et al. Long COVID (post-COVID-19 condition) in children: a modified Delphi process. *Archives of Disease in Childhood* 2022; **107**(7): 674.
