# Supplementary material for: Natural course of health and well-being in non-hospitalised children and young people after testing for SARS-CoV-2: a prospective follow-up study over 12 months
Source: Lancet Reg Health Eur. 2022 Dec 5;25:100554. doi: 10.1016/j.lanepe.2022.100554 (PMC9719829; doi:10.1016/j.lanepe.2022.100554)
Supplement: LANCET STROBE [file mmc2.docx]

STROBE Statement—checklist of items that should be included in reports of observational studies

|  | Item No. | Recommendation | Page  No. | Relevant text from manuscript |
| --- | --- | --- | --- | --- |
| **Title and abstract** | 1 | (*a*) Indicate the study’s design with a commonly used term in the title or the abstract | 3 | Abstract “Methods: A non-hospitalised, national sample of 5,086 (2,909 SARS-COV-2 Positive; 2,177 SARS-COV-2 Negative at baseline) CYP aged 11-17 completed questionnaires 6- and 12-months after PCR-tests between September 2020 and March 2021 confirming SARS-CoV-2 infection (excluding CYP with subsequent (re)infections). SARS-COV-2 Positive CYP were compared to age, sex and geographically-matched test-negative CYP..” |
|  |  | (*b*) Provide in the abstract an informative and balanced summary of what was done and what was found | 3 | See Abstract |
| Introduction | | | |  |
| Background/rationale | 2 | Explain the scientific background and rationale for the investigation being reported | 6 | See “Introduction” |
| Objectives | 3 | State specific objectives, including any prespecified hypotheses | 6 | Introduction, paragraph 3 “We therefore collected longitudinal information on a larger group of CYP at 6- and 12-months post-test and here we describe the within-individual variation in health and well-being 6- and 12-months after testing.” |
| Methods | | | |  |
| Study design | 4 | Present key elements of study design early in the paper | 7 | See paragraphs 1 and 2 under “Methods” |
| Setting | 5 | Describe the setting, locations, and relevant dates, including periods of recruitment, exposure, follow-up, and data collection | 7-9 | For setting, locations, and relevant dates, including periods of recruitment: see paragraphs 1-3 under “Methods”  For exposure, follow-up, and data collection: see “Measures” |
| Participants | 6 | (*a*) *Cohort study*—Give the eligibility criteria, and the sources and methods of selection of participants. Describe methods of follow-up  *Case-control study*—Give the eligibility criteria, and the sources and methods of case ascertainment and control selection. Give the rationale for the choice of cases and controls  *Cross-sectional study*—Give the eligibility criteria, and the sources and methods of selection of participants | 7, 9 | For eligibility criteria, sources and methods of selection of participants: see Supplementary Table 1 and paragraph 2 under measures.  For methods of follow-up: see 2^nd^ paragraph under “Measures” |
|  |  | (*b*) *Cohort study*—For matched studies, give matching criteria and number of exposed and unexposed  *Case-control study*—For matched studies, give matching criteria and the number of controls per case | 7 | For matching criteria: see 1^st^ paragraph under “Methods” |
| Variables | 7 | Clearly define all outcomes, exposures, predictors, potential confounders, and effect modifiers. Give diagnostic criteria, if applicable | 9 | As per objectives (item 3), there are no predictors, potential confounders, and effect modifiers.    For diagnostic criteria of Long COVID: see 2^nd^ paragraph under “Measures” regarding the operationalisation of the research definition of long COVID |
| Data sources/ measurement | 8* | For each variable of interest, give sources of data and details of methods of assessment (measurement). Describe comparability of assessment methods if there is more than one group | 9 | See “Measures” and Supplementary text 1 for questionnaire. |
| Bias | 9 | Describe any efforts to address potential sources of bias | 9 | See paragraph 1 of statistical methods: “We first assessed the representativeness of our analytic sample by comparing their demographic characteristics (sex, age at testing, region of residence, and Index of Multiple Deprivation) to the target population invited 6-months post-test” |
| Study size | 10 | Explain how the study size was arrived at | 7-8 | See paragraphs 2 and 3 of “Methods” |

Continued on next page

| Quantitative variables | 11 | Explain how quantitative variables were handled in the analyses. If applicable, describe which groupings were chosen and why | 9 | See Statistical methods |
| --- | --- | --- | --- | --- |
| Statistical methods | 12 | (*a*) Describe all statistical methods, including those used to control for confounding | 9 | See Statistical methods. |
|  |  | (*b*) Describe any methods used to examine subgroups and interactions | 9 | See Statistical methods |
|  |  | (*c*) Explain how missing data were addressed | NA | No missing data (be design) |
|  |  | (*d*) *Cohort study*—If applicable, explain how loss to follow-up was addressed  *Case-control study*—If applicable, explain how matching of cases and controls was addressed  *Cross-sectional study*—If applicable, describe analytical methods taking account of sampling strategy | NA | No loss to follow up (by design, see Figure 1) |
|  |  | (*e*) Describe any sensitivity analyses | 10 | See “Sensitivity Analysis” |
| Results | | | | |
| Participants | 13* | (a) Report numbers of individuals at each stage of study—eg numbers potentially eligible, examined for eligibility, confirmed eligible, included in the study, completing follow-up, and analysed | 10-11 | See 1^st^ paragraph of results, table 1, figure 1 |
|  |  | (b) Give reasons for non-participation at each stage | Figure 1 | See Figure 1 |
|  |  | (c) Consider use of a flow diagram | Figure 1 | See Figure 1 |
| Descriptive data | 14* | (a) Give characteristics of study participants (eg demographic, clinical, social) and information on exposures and potential confounders | Table 1 | See Table 1 |
|  |  | (b) Indicate number of participants with missing data for each variable of interest | NA | No missing data (be design) |
|  |  | (c) *Cohort study*—Summarise follow-up time (eg, average and total amount) | 10 | See paragraph 1 of “Results” |
| Outcome data | 15* | *Cohort study*—Report numbers of outcome events or summary measures over time | Figures 2-8 | Figures 2-8 |
|  |  | *Case-control study—*Report numbers in each exposure category, or summary measures of exposure |  |  |
|  |  | *Cross-sectional study—*Report numbers of outcome events or summary measures |  |  |
| Main results | 16 | (*a*) Give unadjusted estimates and, if applicable, confounder-adjusted estimates and their precision (eg, 95% confidence interval). Make clear which confounders were adjusted for and why they were included | NA | As per objectives (item 3) no adjusted analyses undertaken. |
|  |  | (*b*) Report category boundaries when continuous variables were categorized | NA |  |
|  |  | (*c*) If relevant, consider translating estimates of relative risk into absolute risk for a meaningful time period | NA |  |

Continued on next page

| Other analyses | 17 | Report other analyses done—eg analyses of subgroups and interactions, and sensitivity analyses |  | See Supplementary Material |
| --- | --- | --- | --- | --- |
| Discussion | | | | |
| Key results | 18 | Summarise key results with reference to study objectives | 13-14 | See Discussion paragraphs 1-3 |
| Limitations | 19 | Discuss limitations of the study, taking into account sources of potential bias or imprecision. Discuss both direction and magnitude of any potential bias | 16-18 | See Discussion paragraph 8 |
| Interpretation | 20 | Give a cautious overall interpretation of results considering objectives, limitations, multiplicity of analyses, results from similar studies, and other relevant evidence | 18 | See Conclusions |
| Generalisability | 21 | Discuss the generalisability (external validity) of the study results | 16 | See Discussion paragraph 7 |
| Other information | |  | | |
| Funding | 22 | Give the source of funding and the role of the funders for the present study and, if applicable, for the original study on which the present article is based | 21 | See Acknowledgments |

*Give information separately for cases and controls in case-control studies and, if applicable, for exposed and unexposed groups in cohort and cross-sectional studies.

**Note:** An Explanation and Elaboration article discusses each checklist item and gives methodological background and published examples of transparent reporting. The STROBE checklist is best used in conjunction with this article (freely available on the Web sites of PLoS Medicine at http://www.plosmedicine.org/, Annals of Internal Medicine at http://www.annals.org/, and Epidemiology at http://www.epidem.com/). Information on the STROBE Initiative is available at www.strobe-statement.org
